# Quantification of food waste per product group along the food supply chain in the European Union: a mass flow analysis

**DOI:** 10.1016/j.resconrec.2019.06.011

**Published:** 2019-10

**Authors:** Carla Caldeira, Valeria De Laurentiis, Sara Corrado, Freija van Holsteijn, Serenella Sala

**Affiliations:** a European Commission-Joint Research Centre, Via Enrico Fermi 2749, I-21027 Ispra, VA, Italy; b VHK BV, Rotterdamseweg 386 B-18, 2629 HG, Delft, the Netherlands

**Keywords:** Food waste, Mass balance, Food groups, By-products, Systematic accounting, Food value chain

## Abstract

In 2015, the United Nations defined the Sustainable Development Goals (SDG), which include a target (12.3) on food waste. The target requires “by 2030, to halve per capita global food waste at the retail and consumer levels and to reduce food losses along production and supply chains, including post-harvest losses”. The target has increased awareness about the food waste problem and boosted research in food waste quantification. Nevertheless, there is a lack of studies that adopt a systematic approach to account for food waste providing disaggregated values per food supply chain stage and per food groups. Such an approach could support policy makers in prioritizing interventions for food waste reduction. To fill this gap, this paper presents a high-level top-down approach to food waste accounting in the European Union. The study aims to support the understanding of the mass flows associated with food production, consumption, and waste, addressing different food groups along the food supply chain. The method for accountin is the mass flow analysis. According to the results, cereals, fruit, and vegetables as the food groups are responsible for the highest amount of food waste, and the consumption stage to be responsible for the largest share of food waste for most food groups. This work highlights the need for further primary research on food waste generation in the EU. Ultimately, this would allow to robustly estimate the food waste generated at EU level, and establishing a more accurate baseline to track the progress towards SDG target 12.3.

## Highlights

• A systematized food waste accounting at macro scale level was developed for Europe.
• The accounting follows Mass Flow Analysis concepts, ensuring closed mass balance.
• Disaggregated values per food supply chain stages and per food groups are presented.
• Hotspots and food groups with higher share of food waste were identified.
• The stage contributing the most to food waste varies depending on the food group.

## 1 Introduction

The foreseen growth of the world’s population will increase significantly the pressure on natural resources to respond to food needs. To achieve food security for all, in a context of constrained resources and changing climate, without further compromising ecosystems quality and biodiversity, a multidimensional and integrated global strategy is necessary (Godfray et al., 2010). An essential action is to increase the efficiency of the entire food supply chain (FSC), which includes reducing the amount of food wasted (Foley et al., 2011; Foresight, 2011). Food waste is a major global challenge not only from an ethical and social point of view, but also from environmental and economic ones. Furthermore, it represents an inefficient use of the scarce resources used to produce it, such as land and water (FAO, 2013).

Worldwide, efforts and initiatives are being implemented and developed to address food waste. In 2015, the United Nations defined the Sustainable Development Goal (SDG) 12 to “Ensure sustainable consumption and production patterns” within which, target 12.3 is referring to food waste: “By 2030, halve per capita global food waste at the retail and consumer levels and reduce food losses along production and supply chains, including post-harvest losses” (UN, 2015). The European Commission (EC) committed to achieve the SDG target in the European Circular Economy Action Plan, defining food waste as a priority area (European Commission, 2015). Moreover, the EC amended the Waste Framework Directive 2008/98/EC (WFD) setting as obligatory the monitoring and reporting on food waste by Member States (MSs) to: (i) establish a baseline to monitor the achievement of food waste reduction targets; and (ii) help in the identification of relevant food waste streams to be valorised in a circular economy perspective (European Commission, 2018).

An essential aspect to design efficient and effective policies to reduce food waste along the FSC is to know how much and where the food is being wasted. This allows to: (i) establish a baseline against whichprogress towards the targets can be measured, (ii) monitor food waste generation over time, (iii) identify hotspots along the FSC for food waste reduction, and (iv) identify potential food waste valorisation pathways. However, food waste accounting is still at an early stage of development and a consolidated framework for food waste quantification in the European Union (EU) is still an open challenge (Caldeira et al., 2017; Corrado et al., 2019; Xue et al., 2017).

Studies can be found in the literature that present estimates of food waste generated at EU level using various data sources, such as statistics and direct surveys (Alexander et al., 2017; Bräutigam et al., 2014; EUROSTAT, 2017; FUSIONS, 2016; Gustavsson et al., 2011; Kemna et al., 2017; Monier et al., 2010; Porter et al., 2016; Vanham et al., 2015). A review of the studies focusing on the EU and global scale shows that results for EU range from 158 to 298 kg/year/capita (Corrado and Sala, 2018). The discrepancies amongst the reported estimates are due to differences in: scope of the studies, system boundaries, aims and methods, adopted definitions, and inherent accounting problems (Corrado and Sala, 2018).

In order to prioritise interventions for food waste reduction, a systematized approach to food waste accounting at macro scale level (e.g. national, continental, or global level) is necessary. This paper contributes to fill this gap, focusing on the accounting of food waste at EU scale, by adopting life cycle thinking, namely with a breakdown per stages of the FSC as well as per product groups. The approach developed is based on the theoretical framework of Mass Flow Analysis (MFA) that “is a systematic assessment of the flows and stocks of materials within a system defined in space and time” (Brunner and Rechberger, 2004), ensuring a closed mass balance from stage to stage. MFA has proven to be useful in the support of waste management and recycling policies by allowing the mapping and quantification of flows that are to be managed and recycled (Allesch and Brunner, 2015; Moriguchi and Hashimoto, 2016).

Very few studies exist that make use of MFA to account for food waste. Beretta et al. (2013) followed this approach in their study on food losses quantification in Switzerland to determine mass and energy flow analysis and in the quantification of food efficiency. Betz et al. (2015) used MFA to demonstrate the quantitative (in kg) and monetary (cost price in CHF) losses caused by food wasted at each step of the supply chain of two food service companies in Switzerland. Kemna et al. (2017) accounted for food waste at EU level using a combination of various statistical data sources and scientific literature.

This work builds on Kemna et al. (2017) by additionally providing a systematized approach to perform food waste accounting at EU level based on MFA, including a detailed compilation of coefficients that can be used to fill data gaps when modelling food waste flows. Moreover, it presents a breakdown per FSC stage - primary production (PP), processing and manufacturing (P&M), distribution and retail (D&R), and consumption both at household and food service sectors - of different food groups, enabling the identification of hotspots. It also considers circular flows of food removed from the FSC to be used to produce other products such as animal feed, which are here referred to as by-products. Such a high-level top-down approach helps understanding the mass flows associated with food production, consumption, and waste and can be considered a necessary complement to direct data measurements, such as waste composition analyses, surveys, and diaries. Moreover, it can be used as a starting point when no specific data exists. A comparison of the results obtained with existing studies accounting for food waste at EU level was also performed.

## 2 Material and methods

This section presents an overview of the approach followed including: the definition of food waste used (Section 2.1); the scope of the study (i.e. geographical and temporal), the system boundaries, the food groups considered (Section 2.2); the main data sources used (Section 2.3); the accounting methodology (Section 2.4); and the uncertainty assessment (Section 2.5).

### 2.1 Food waste definition

This study follows the FUSIONS (a EU-funded project to support MSs in measuring food waste) definitional framework which is consistent with the principles of the Food Loss and Waste Standard, a global standard providing requirements and guidance for quantifying and reporting on food waste (FLW Protocol, 2016). FUSIONS defines food waste as: fractions of ‘food and inedible parts of food removed from the food supply chain’ to be recovered or disposed (including: composted, crops ploughed in/not harvested, anaerobic digestion, bioenergy production, co-generation, incineration, disposal to sewer, landfill or discarded to sea) (FUSIONS, 2014).

Note that, according to the FUSIONS framework and the WFD, food removed from the food supply chain to be valorized as for example animal feed is not considered as food waste. Therefore, in this study, these flows were accounted for as by-products.

### 2.2 Scope of the study: geographical and temporal aspects, system boundaries, and food product groups

This study accounted for food waste generated in the EU in the year 2011. In order to model food flows in the EU, the ideal approach would be to operate at MS level and then calculate the aggregated flows at EU level. This is because large differences exist between MSs in food consumption and food waste generation due to differences in e.g. eating cultures, habits and waste collection systems (Thyberg and Tonjes, 2016; Vanham et al., 2015). Nevertheless, since robust and representative data on food waste are scarce and current studies have been conducted only in a few MSs, it was decided not to differentiate between countries but to focus on EU level.

To come to a complete picture of the food chain, a closed mass-balance approach was applied for the major EU food groups: sugar beets, oil crops, potatoes, vegetables, fruit, cereals, meat, fish, dairy, and eggs. Besides quantifying the food waste generated at each stage of the FSC, this study estimated the flows of food products and by-products (i.e. used for animal feed and other non-food uses) from PP, P&M, D&R, and end-user level where food is consumed at home and at food services (e.g. restaurants, canteens, hospitals). According to the FUSIONS theoretical framework, the starting points of the food supply chain at primary production are crops mature for harvest, animals ready for slaughter, wild fish caught, milk drawn from animals and eggs laid (FUSIONS, 2014). Trade data were included at EU-level covering only extra-EU imports and exports.

### 2.3 Data sources

The main statistical data sources used were: (i) the Commodity Balance Sheets (CBS) (FAO, 2011a) (FAO, 2011b) to obtain the production, stocks, supply and non-food uses of food per product group, (ii) the FAO trade statistics for crops and livestock products, to obtain values of extra-EU imports and exports (FAO, 2011c), and (iii) Eurostat data on trade and production of manufactured products, also known as Prodcom (EUROSTAT, 2011). However, these data sources may be affected by a number of limitations, as part of the data might be missing or be reported with years of delay due to e.g. confidentiality reasons. Besides, Prodcom data only include manufactured products without specifying their destination. Consequently, the possibility of double counting of products cannot be ruled out.

Food waste coefficients (i.e. the percentage of a flow entering a certain stage of the FSC that is wasted) taken from the literature were used to quantify the amount of waste generated at the steps of the FSC where there was a lack of statistical data sources to calculate the waste flows with mass balances. This was the case at PP, D&R and at consumption stages. The process followed to select the food waste coefficients builds on the literature review developed by Xue et al. (2017). Preference was given to studies based on primary data or a combination of modelling and statistical data, developed either at EU level or in an EU member state. Please refer to the supplementary information (SI) for further details on the selection process and on the coefficients used.

In addition, conversion factors taken from the literature were used to transform manufactured products into their raw material equivalents (e.g. FAO, 2003, 1990). One should mention that conversion factors for cooking/preparation were not considered when estimating food waste at consumer level, because it was not possible to know the share of food being thrown away before and after cooking. Although this assumption might influence the quantification of food waste at consumption stage, a study conducted by WRAP (2014) demonstrated that the net changes in the weight of products due to cooking and preparation are limited as certain food items gain weight when cooked while others lose weight.

Finally, EU consumption statistics (EEA, 2017) based on nutrition surveys were used to crosscheck the amount of food consumed in the EU resulting from the accounting exercise. Although their scope is to establish the dietary habits of citizens, these studies could be used in combination with food production statistics to validate the accounting of food waste. Nevertheless, when using this data one should be aware that this type of data collection may be uncertain because people tend to underestimate their food intake (Leclercq et al., 2009).

### 2.4 Accounting methodology

**Fig. 1:**
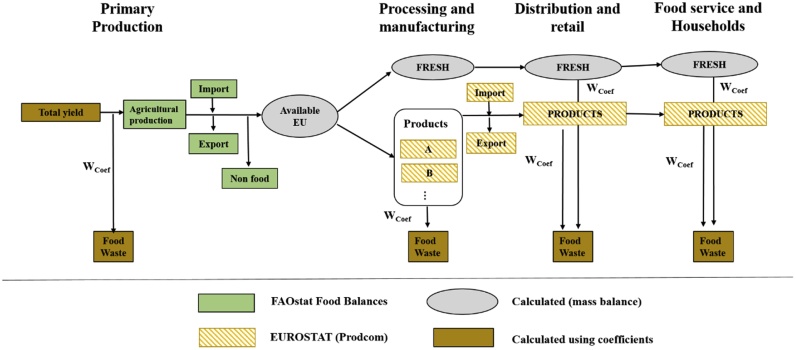
Accounting approach and main sources of data used to calculate food waste flows. Due to the specificities of each production process and data availability, the accounting approach adopted at processing and manufacturing was tailored to each food group. The example provided in this figure is illustrative for the following food groups: fish, fruit, vegetables and potatoes. W_Coef_-waste coefficients.

The food waste accounting methodology is based on the principles of life cycle thinking and MFA. Fig. 1 provides a general scheme of the accounting approach adopted, by presenting for each step of the FSC how the flows of food waste were calculated from the various data sources used. According to the availability of data, the food waste flows were calculated following two approaches: when all the inputs (i.e. amounts entering a FSC stage) and the outputs (i.e. amounts leaving a FSC stage) were known, a mass balance approach was used to calculate the flows of food waste and by-products, following the principle of mass conservation. This was the approach adopted when calculating the waste and by-products generated at P&M of sugar beet, oil crops, and partially cereals. When only a part of the inputs and/or of the outputs were known, coefficients from the literature were used instead. This was the case for food waste generated at PP, D&R, consumption and P&M for the remaining food groups, where the data were not sufficient to perform a mass balance. The reader is referred to the SI for more details. The starting point of the accounting is the primary production (excluding pre-harvest losses) obtained from the CBS (FAO, 2011a). From this quantity, the “total yield” (i.e. including pre-harvest losses) was calculated based on coefficients from the literature. Trade flows were included at two points in the supply chain: (i) the extra-EU imports and exports of raw materials, taken from the FAOSTAT trade statistics for crops and livestock products (FAO, 2011c), and (ii) the extra-EU imports and exports of manufactured products, taken from Prodcom (EUROSTAT, 2011).

The food flow accounting exercise used the following principles:

• All flows are expressed according to the real weight of commodities at each stage of the food supply chain (e.g. tonnes of orange juice imported).
• The accounting exercise is based on a closed system where inputs equals outputs. Internal recycling flows are shown as by-products going to animal feed.
• A territorial approach is adopted, i.e. only the food waste generated in the EU is accounted for and not the food waste embedded in the net imports of raw and manufactured products.
• The outputs of the accounting system are the end-use of products, exports (of raw materials and manufactured products separately), flows to non-food industry (of vegetal and animal inputs separately), food going to animal feed, food consumed, and food waste.
• Other material flows not explicitly taken into account in the food flow accounting are water for irrigation and drinking water, fertilizers, manure, pesticides, and energy carriers. These flows are not subject of this study.
• At consumption stage (households and food services) a split between edible food waste and the associated inedible parts is provided.

The split between the edible and inedible components of food waste was not conducted at the remaining stages of the supply chain. At PP and D&R the reason for not providing such a split is the fact that food items are generally wasted as a whole (e.g. an apple left on field), therefore an estimation of the edible component does not provide additional information on the share of food waste that could be avoided by implementing food waste prevention strategies. Nevertheless, the edible component could be calculated from the total food waste provided by using food composition databases (e.g. Rimestad et al., 2017). At P&M such split was not provided due to a lack of data. Nevertheless, as production inefficiencies were not considered, most of the food waste estimated at this stage is inedible.

A brief description on how food flows were modelled in each stage follows.

#### 2.4.1 Primary production (PP)

The quantification of food waste generated at PP has received very little attention and a limited number of studies exist that address this issue (Beausang et al., 2017). Those that exist are focused at country level (Redlingshöfer et al., 2017) and typically cover a limited number of food products (Franke et al., 2016; Roels and van Gijseghem, 2017). A study performed by Hartikainen et al. (2018) presented an estimation of food waste at PP in four Nordic countries - Finland, Sweden, Norway, and Denmark - based on existing literature and case studies, providing the most comprehensive (in terms of food groups covered) set of coefficients to determine food waste at primary production that is in accordance with the FUSIONS framework. Therefore, these coefficients were adopted in this study to calculate food waste at PP. Nevertheless, one should keep it mind that the countries where these data were collected are not representative of the EU.

Hartikainen et al. (2018) provided two sets of coefficients. The first set, sf_pp,i_ (side flow percentages) enables to calculate what they defined as “side flow”: this term is used by the authors as a synonym of food waste and it refers, similarly to the coefficients of the FAO framework (Gustavsson et al., 2011), to the flows of primary products meant to be eaten by humans but that never entered the next stage of the FSC (including food waste used for the production of animal feed). The second set of coefficients, ffw_pp,i_ (FUSIONS food waste percentage), enables to calculate the food waste at PP according to the FUSIONS definition (i.e. excluding food waste used for animal feed). As the production statistics (e.g. the CBS) do not include food waste generated at primary production, the sf_pp,i_ coefficients were used to calculate the total yield for each product group, according to Eq. (1). Then the food waste generated (FW_PP,i_), in line with the FUSIONS definition, was calculated by means of Eq. (2). Both coefficients sf_pp,i_ and ffw_pp,i_ are based on averages or on singular figures (depending on the amount of data available) for losses and waste derived from questionnaires, as presented in Hartikainen et al. (2018). The coefficients (average value) used for each food group (i) obtained from Hartikainen et al. (2018) can be found in the SI (table S1).

(1) $$\text{To}\text{t}\text{a}\text{l}\;\text{y}\text{i}\text{e}\text{l}\text{d}=\;\frac{\text{P}}{\left(1-\text{s}{\text{f}}_{\text{P}\text{P},\text{i}}\right)}$$

(2) $$\text{F}{\text{W}}_{\text{P}\text{P},\;\text{i}}=\;\frac{\text{P}}{(1-\;\text{s}{\text{f}}_{\text{P}\text{P},\text{i}}\text{)}}\text{*}\text{f}\text{f}{\text{w}}_{\text{p}\text{p},\text{i}}\;$$

where P is the food production amount obtained from the CBS.

The underlying assumption made when calculating flows of waste and by-products at primary production, was that the food waste at primary production would only be calculated for the portion of crops harvested for food production. In other words, the waste at primary production deriving from the harvesting of oil crops used for biofuels and from cereals produced for animal feed was not taken into account.

#### 2.4.2 Processing and manufacturing (P&M)

The amount of food entering the P&M stage was calculated through a mass balance (production plus imports minus exports and other uses, obtained from FAO, 2011a, 2011b, 2011c). From here, food is either destined for fresh consumption or enters a series of manufacturing processes, affecting the initial mass of each product.

As illustrated in Fig. 1, the following procedure was used to calculate food waste and by-products at this stage:

- the amount of manufactured food products for each product group was taken from Prodcom;
- their fresh equivalent mass was calculated using conversion coefficients from the literature (provided in SI, tables S4 and S7);
- the processing waste and by-products were obtained by multiplying the amount expressed as fresh equivalent by waste coefficients (equal to the inedible fraction of the fresh product, provided in SI, tables S5 and S8) taken from the literature.

Added and/or evaporated water and added ingredients were accounted for within the conversion coefficients. Processing waste and by-products include, amongst others, inedible parts like peels, bones, and scrapings. In some cases these are used for non-food purposes (e.g. cosmetics, glue, pet-food) and therefore are considered to be by-products and are accounted for separately from the food waste flows.

Due to the specificities of each production process and data availability, the procedure above was tailored to each food group. A detailed explanation on how the food waste was calculated for each food group and the corresponding coefficients used can be found in the SI (Section 2).

In this study, different processing technologies and efficiencies of production processes were not taken into consideration.

Finally, the amount of each food group leaving this FSC stage as fresh (for products consumed fresh, such as fruit and vegetables) was calculated as the difference between the total amount of each product entering this stage and the raw equivalent amount required to obtain the manufactured products reported.

#### 2.4.3 Distribution and retail (D&R)

At the D&R stage, food waste occurs from both fresh and manufactured products. Food waste was calculated using waste coefficients that refer to the waste generated at D&R for fresh and manufactured products obtained from the literature (as explained in Section 2.3) multiplied by the amount of products to be distributed. The coefficients used can be found in Table S9 of the SI.

#### 2.4.4 Consumption: household and food service

At the consumption stage, food waste occurs at households and food services. A split was made between consumption in households (85%) and in food services (15%), based on a study performed by Beretta et al. (2013). Food waste from households and the food service sector was then calculated using waste coefficients for fresh and manufactured products obtained through a literature review (Section 2.3). The coefficients used can be found in Table S9 of the SI. The remaining products were considered to be eaten. These amounts were compared with the average food consumption data from a study published by the European Environment Agency (EEA) (EEA, 2017). At this stage, a differentiation was made between edible food waste and the associated inedible parts, in order to highlight the share of food waste generated by households and food services that could be reduced by applying targeted prevention strategies. To this end, the edible food waste was calculated from the amount of food entering the consumption stage, using coefficients that refer to the edible food waste generated by households and food services, taken from the literature. The full list of coefficients used is provided in table S10 of the SI. For certain products (e.g. pasta) all the food waste calculated at consumption stage is edible (as there are no inedible parts), and for this reason no further calculation was performed.

#### 2.4.5 Food waste and by-products embedded in imports

To understand how the adoption of a consumption-based approach would affect the results of this study, we calculated the amount of food waste and by-products embedded in traded food. The amount of food waste and by-products associated with EU food consumption and generated outside EU boundaries, i.e. food waste and by-products embedded in traded food, was calculated by multiplying food waste coefficients by the net trade, i.e. obtained as imported minus exported amounts, at PP and P&M. Here, we adopted the same food waste coefficients as for the EU. In cases in which coefficients for P&M were not explicitly reported, e.g. cereals, they were gathered from the calculations done for the EU by calculating for each food group the ratio between the sum of waste and by-products generated and the amount of food entering the stage. The split between food waste and by-products depends on the place where the food was produced and was not considered due to lack of information.

### 2.5 Uncertainty assessment

Food waste accounting entails several sources of uncertainty including for example, systematic errors (bias), methodological errors, data processing errors, conversion from amounts to weight, model uncertainty, or assumptions (FLW Protocol, 2016). In the case of mass balance methods, the uncertainty associated with the data used to estimate the amounts of food waste will impact the uncertainty of the results (FLW Protocol, 2016). FAO and EUROSTAT data are subject to uncertainties and limitations (Bräutigam et al., 2014) and most of the coefficients used in this study are not representative for the EU.

The waste coefficients used in this work were collected from eight studies. Six studies (Mena et al., 2014; WRAP, 2009; WRAP, 2011; WRAP, 2014; Willersinn et al. 2015; Hartikainen et al. 2018) based their results on direct measurements (e.g. waste composition analysis, kitchen diaries, surveys, interviews). Beretta et al. (2013) used a mix of direct measurement (i.e. interviews and records collected by food manufacturers and retailers) and literature sources, and De Laurentiis et al. (2018) adopted a modelling approach based on several literature sources, to derive coefficients for avoidable and unavoidable waste of fresh fruit and vegetables representative for the EU. Of the studies that used direct measurement (at least partially), the field work was conducted in Switzerland (for 53% of the coefficients used), in the UK (for 37% of the coefficients used), and in the Nordic countries - Finland, Sweden, Denmark and Norway (for 11% of the coefficients used). Field work was conducted between the years 2007 and 2015, but the most represented years are 2011 and 2012 (as 80% of the coefficients used are based on field work from those two years). Waste coefficients were used to determine food waste amounts at primary production, distribution and consumption. Considering that these stages all together are responsible for a high share of the food waste generated, we performed an analysis of the influence of the uncertainty associated with the waste coefficients on the final results. First, we used a semi-quantitative approached based on the use of a pedigree matrix to determine the uncertainty factor associated with each waste coefficient (Beretta et al., 2013). In this approach, the data is assessed according to its reliability, completeness, temporal, geographical and further technological correlation, and sample size. More information on the calculation of the uncertainty factors is provided in the SI (section 6.1). Then, a Monte Carlo simulation was performed with 10 000 runs to analyse the uncertainty of the results. To analyse which coefficients contribute the most to the variation, the contribution to the variance of the waste coefficients was calculated using global sensitivity analysis. Spearman’s rank correlation coefficients (Hamby, 1994) was used as the measure of sensitivity that allows ranking different uncertain inputs based on their level of contributions to the variance of modelled output. Since we had no information on the probability profile of the waste coefficients, we assumed a uniform distribution; therefore assuming that all coefficient values are equally likely (Gregory et al., 2013).

## 3 Results and discussion

The results of the study are presented in Section 3.1 followed by a comparison with other studies (Section 3.2), and a discussion on the strengths and limitations of this approach and of future research needs to improve the accounting of food waste at EU level (Section 3.3).

### 3.1 Overall food waste flows in the EU

**Fig. 2:**
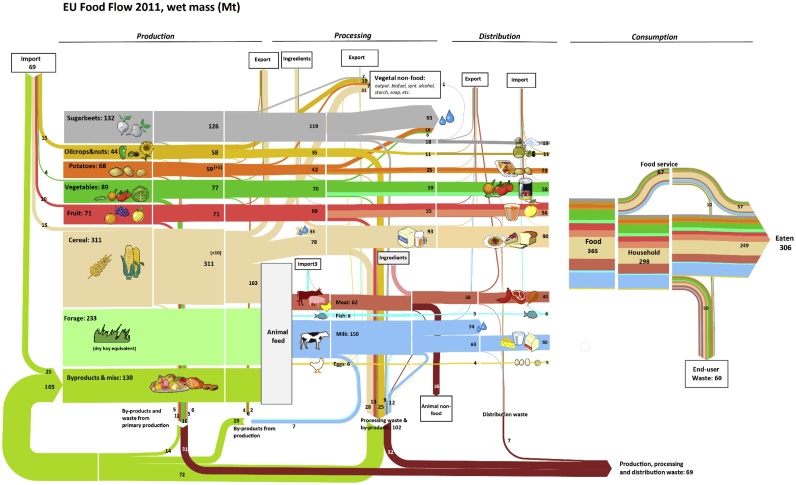
Sankey diagram of the product flows and food waste generated along the Food Supply Chain. The diagram contains feed and food flows, excluding soft drinks, mineral waters and some non-perishable foodstuffs (salt, coffee, etc.). Modified from Kemna et al. (2017). The numbers in brackets refer to amounts used as seeds.

A complete overview of the mass flow analysis of EU food and waste flows performed is provided in Fig. 2 in the form of a Sankey diagram. Here, the flows of each product group are visualised, starting from the amounts of food produced at primary production and ending at consumption. All the flows entering the supply chain as imports and leaving it as exports, by-products to non-food, by-products to animal feed, food waste, and food consumed by humans are represented. Background data used to build the Sankey diagram are provided in SI tables S11 and S12.

**Fig. 3:**
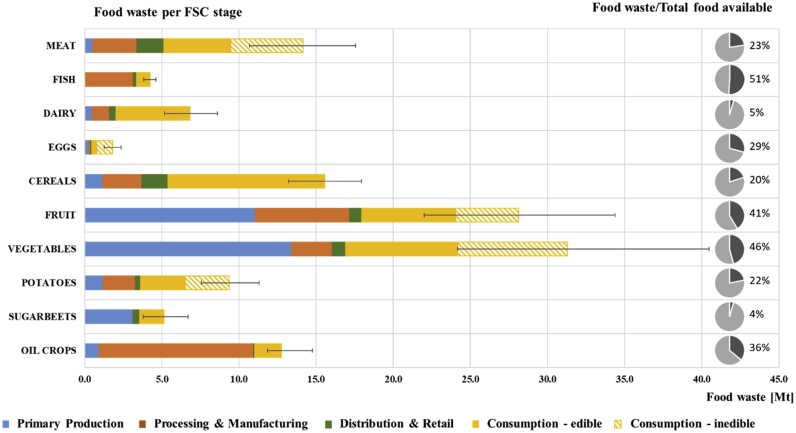
Left: Total food waste amount (including edible and inedible components) calculated along the FSC for each food group. The error bars represent the range between the minimum and maximum value of the food waste calculated for each group due to the variation assumed in the coefficients used to calculate food waste at PP, D&R and consumption. Right: percentage of food waste (dark grey) out of the total food available.

Table 1 and Fig. 3 illustrate the amounts of food waste generated for each food group at each FSC stage. Furthermore, a comparison between the food waste generated and the quantity of each product group entering the FSC (defined as ‘EU available’ and calculated as production plus import minus exports of primary commodities minus non-food uses) is provided in Table 1. Food used for animal feed and non-food purposes is presented in Table 2.

**Table 1:** Food available in the EU, and food waste calculated for each food group and FSC stage for 2011.

Food groups	EU available (Mt)	Food Waste (Mt)					
		Primary Production	Processing & Manufacturing	Retail & Distribution	Consumption		Total FW
					Households	Food services	
**Meat**	61.7	0.5	2.9	1.7	7.3	1.7	14.2
**Fish**	8.2	0.0	3.1	0.2	0.5	0.3	4.2
**Dairy**	150.2	0.5	1.1	0.4	4.2	0.6	6.8
**Eggs**	6.2	0.3	0.1	0.1	1.1	0.3	1.8
**Cereals**	78.2	1.2	2.5	1.7	8.0	2.2	15.6
**Fruit**	67.9	11.1	6.1	0.8	8.6	1.5	28.1
**Vegetables**	68.5	13.4	2.6	0.9	12.2	2.2	31.3
**Potatoes**	42.8	1.2	2.1	0.3	4.9	0.8	9.4
**Sugar beets**	118.7	3.1	0.0	0.4	1.3	0.3	5.1
**Oil Crops**	35.4	0.9	10.0	0.1	1.4	0.3	12.7
**TOTAL**	**637.8**	**32.2**	**30.6**	**6.7**	**49.6**	**10.3**	**129.2**

**Table 2:** Amount of each commodity used for animal feed and non-food purposes at primary production and processing and manufacturing.

	Primary Production		Processing and Manufacturing	
	Feed (Mt)	Non Food (Mt)	Feed (Mt)	Non Food (Mt)
**Meat**	—	—	—	26.4
**Fish**	1.8	0.26	1.5	—
**Dairy**	4.6	0.36	11.4	—
**Eggs**	—	0.04	—	0.3
**Cereals**	167.3	30.7	25.6	—
**Fruit**	0.2	0.1	7.5	—
**Vegetables**	7.4	0.1	2.6	—
**Potatoes**	9.1	6.7	—	—
**Sugarbeet**	3.3	6.8	8.5	—
**Oilcrops**	2.3	18.9	14.6	—
**Total**	**195.9**	**64.0**	**71.7**	**26.6**

A total input of around 638 Mt primary food commodities results in approximately 129 Mt of food waste generated along the FSC. The largest share is generated at the consumption level, equal to 60 Mt (representing 46% of the total food waste), followed by PP (25%), P&M (24%), and D&R (5%).

The food groups presenting the highest amount of food waste (considering the total food waste generated in all the supply chain stages) are fruit and vegetables. Eggs and fish present the lowest amounts, but these products are also the ones being consumed in smaller quantities (Table 1). Nevertheless, in the case of fish, the share of food waste out of the total food available for these products is higher than the one of fruit and vegetables (Fig. 3).

It is a logical argument that the food groups that are produced and consumed in higher quantities present higher food waste amounts. Nevertheless, as shown in Fig. 3, there are some exceptions to this rationale. For example, fruit, vegetables, and cereals present similar amounts entering the FSC (67.9 Mt, 68.5 Mt and 78.2 Mt) but the food waste of cereals (15.6 Mt, 20% of the amount entering the FSC) is almost half of the fruit and vegetable food waste (28.1 Mt and 31.3 Mt, around 45% of the amount entering the FSC). This is due to the higher share of inedible parts in these products (which contribute to 40% and 49% of the waste flows at consumption level of fruit and vegetables, respectively) and also to the higher perishability of fruit and vegetables compared to some cereal-based products (e.g. pasta and rice). Also, fruit and vegetables represent together 76% of the waste at primary production, as the coefficients used for these products are much higher than the others.

The consumption stage is responsible for the largest share of food waste generation for most food groups, as illustrated by Fig. 3. This is the case for meat, dairy, eggs, cereals, vegetables, and potatoes. Instead, for the fish supply chain the highest share of food waste (73%) is generated at the P&M stage. This is because the fish processing originates a significant amount of food waste that is not usually valorised (Jackson and Newton, 2016) in opposition to what happens for example with the meat. Although the meat P&M stage also generates a large amount of inedible parts, such as bones, bloods, inedible organs, and skin, a high share of them (assumed to be equal to the 80% of waste and by-products generated) is being used in other industries (Pearson and Dutson, 2013) and therefore it is not accounted as waste. Furthermore, only 1% of waste was generated at the PP of fish; however, fish discards during fishing are excluded from this figure. According to Davies et al. (2009) by-catches represents around 40% of global marine catches. A high share of food waste at the P&M stage is observed also for oil crops (79%), related to the processing of olive oil. In the case of sugar beets, the largest share of food waste is generated at PP, as all by-products from the processing of sugar beets are assumed to be used for the production of animal feed. This is also the case for fruit, where 39% of the food waste is generated at PP. The reader is referred to Figure S2 of the SI for a clearer visualisation of the relative contribution of the stages of the FSC to the total amount of food waste generated, across food groups.

A split between edible food waste and the associated inedible parts is provided for the consumption stage (Fig. 3). This illustrates how, for certain food groups, the inedible fraction of products at the point of purchase can represent a significant share of the food waste generated by households and food services. Such aspect should be kept in mind when considering future patterns of food waste generation, as mentioned in De Laurentiis et al. (2018), where the concept of “waste floor” was introduced. This was described as a level of household food waste under which it is not possible to go unless consumption patterns change (e.g. an increase in the consumption of frozen instead of fresh vegetables). Nevertheless, for most food groups the largest share of food waste generated at this stage is edible, demonstrating that there is a large potential for food waste reduction through the implementation of targeted prevention strategies towards consumers.

As mentioned in Section 2.5, an uncertainty assessment was performed considering a variation associated with the waste coefficients used to calculate food waste at PP, D&R and consumption stages. The uncertainty associated with the waste coefficients used at primary production was estimated as 27%, at retail it varied from 21% to 43%, at household from 21% to 35%, and at the food service 24% and 80%, depending on the food commodity. Detailed information on the waste coefficients uncertainty estimated is presented in SI tables S17 to S20. Is worth to highlight that the uncertainty associated with the coefficients may vary depending on the quality of the study. The total amount of food waste obtained ranges between 119 and 145 Mt. The variation of the food waste amount calculated for each food group is presented in Fig. 3. The largest variation is observed for fruit and vegetables because the waste coefficients for these food groups are higher (SI tables S1 and S9) and also because for these food groups, the largest share of waste is generated at PP and consumption stages (where food waste coefficients have been used). For oil crops and fish, the variation is small because for these food groups the largest share of waste is originated at P&M, where instead a mass balance approach and a mixed approach based on conversion coefficients (not subject to uncertainty assessment) were adopted, respectively.

The contribution to the variance shows that the waste coefficients contributing the most to the uncertainty of the total waste values are the waste coefficients for vegetables and fruit at primary production, contributing to 29% and 19% of the variance, and the waste coefficient for fresh vegetables at household level, contributing to 13% of the variance. The contribution to the variance for all the groups is presented in the SI (table S21).

#### 3.1.1 Comparison with food consumption data

As mentioned in Section 2.4, the mass balance exercise ends at the consumption stage having as one of the outputs the amount of food consumed. To check these values, this amount was compared with the average food consumption data reported in EEA (2017). The deviation of the values calculated in this study from the ones reported by the EEA were calculated; a positive deviation value means that this study obtained a higher value of consumption for that specific food group compared to the one obtained by the EEA, while negative deviation values mean the opposite. The food consumption calculated in this study is generally higher than the values from the EEA. The most significant differences were observed for sugar and oil crops, where the consumption values found in this study were higher than the ones found by the EEA by 70% and 49%, respectively. Negative deviations were obtained only for fruit (-30%) and potatoes (-21%). Since these products are perceived as 'healthy', there may be a tendency for surveys to overestimate them (via social desirability bias), which would explain the negative deviation encountered. A table with all the results of this comparison is presented in Section 5 (table S13) of the SI.

A possible explanation for the higher values of consumed food obtained in this study compared to the EEA data, may be that there is a higher amount of food waste that was not captured. On the other hand, as the values from the EEA study are based on consumer surveys, it is possible that they are underestimated, as it is known that this type of data collection may be uncertain because people tend to underestimate their food intake (Leclercq et al., 2009). Additionally, a higher level of uncertainty is to be expected for products that are mainly used as ingredients in processed food (e.g. sugar used for confectionary and biscuits), as nutritional surveys tend to classify the products as they are consumed, and therefore assumptions are made subsequently to estimate the intake of each ingredient.

#### 3.1.2 Animal feed and non-food destinations

The amount of each commodity used for animal feed and for non-food purposes is presented in Table 2. The reader is referred to Sections 1 and 2 of the SI for more information on how these flows were calculated. It is worth to recall that these flows are not considered food waste according to the food waste definition used in this paper but were accounted for completeness. At PP, of the total quantity used as animal feed, approximately 93% are commodities (mostly cereals) produced for animal feed purposes, and reported as such in the CBS (FAO, 2011a), and the remaining 7% are commodities originally produced for human consumption that were instead used to produce animal feed, calculated according to Hartikainen et al. (2018). Additionally, 71.7 Mt of by-products from the food processing industry are used for the production of animal feed. The largest contributors are: residuals from the milling of cereals, oil cake (deriving from the production of vegetable oils), and whey (from the production of cheese). The largest share of flows leaving the FSC to be used for non-food purposes is taken by cereals from the PP stage (34%) and meat originated at P&M (29%). The latter includes animal fats, hides and skin, blood and inedible organs. The values presented in Table2 are likely to be an under/overestimation of reality, as little information was available on the share of by-products generated by the food processing industry that are valorised instead of being treated as waste.

### 3.2 Comparison with other studies

A comparison of the results obtained in this study with other studies reporting food waste generated at European level per FSC stage is presented in Table 3. Figures are expressed in kg of food waste generated per capita per year, using the EU population in 2011 obtained from EUROSTAT. The studies selected follow the FUSIONS framework meaning that both edible and inedible food waste is accounted for and that food used for animal feed is not considered food waste (FUSIONS, 2016; Kemna et al., 2017; Monier et al., 2010). The values obtained in this study for the D&R stage, 13 kg/year/capita, and for the consumption stage, 119 kg/year/capita, are within the range of values presented in the other studies varying between 9 and 34 kg/year/capita for D&R, and between 101 and 168 kg/year/capita for consumption. For the P&M stage the amount of food waste estimated in this study is similar to the amounts reported by Monier et al. (2010) and Kemna et al. (2017) but about 50% higher than the value reported in FUSIONS (2016). The major discrepancies are observed for the food waste at PP, that is either not considered (Monier et al., 2010) or results in significantly lower values, i.e. 18 kg/year/capita (FUSIONS, 2016), and 26 kg/year/capita (Kemna et al., 2017) compared to those reported in this study (equal to 64 kg/year/capita). It is important to highlight that the methodological approach and data sources used in Monier et al. (2010) and FUSIONS (2016) differ significantly from those adopted in this study, explaining the variation in the results. Regarding the study by Kemna et al. (2017), the methodological approach adopted is similar to the one of this current study. However, the refinement of the approach and of the coefficients introduced in this work can explain the differences encountered. For a more detailed comparison between the approaches adopted in the three studies presented in Table 3, the reader is referred to Corrado and Sala (2018).

**Table 3:** Comparison of the food waste obtained in this study with results from other studies.

Source	Year	Geographic area	Total Amount (Mt/y)	Primary Production (Kg/y/capita)	Processing & Manufacturing (Kg/y/capita)	Distribution & Retail (kg/y/capita)	Consumption (kg/y/capita)	Total Amount (kg/y/capita)
Monier et al. (2010)	2006	EU27	89	–	70	9	101	**180**
FUSIONS (2016)	2012	EU28	88	18	33	9	113	**173**
Kemna et al. (2017)	2011	EU28	145	26	62	34	168	**290**
**This study**	**2011**	**EU28**	**129**	**64**	**61**	**13**	**119**	**257**

In order to compare the generation of food waste per capita across different countries/continents a consumption-based rather than territorial-based approach should be used. Otherwise, a country importing a significant share of manufactured products would result as generating less food waste per capita than a country that exports large amounts of processed products.

To understand how the adoption of a consumption-based approach would affect the results of this study, we calculated the amount of food waste and by-products embedded in traded food, which resulted to be very low compared to the food waste locally produced in the EU (less than 4 Mt). This is to be expected, as the overall net trade of food products to the EU is small compared to the food consumed, whereas the picture may change when referring to single EU countries, in which the share of traded food products is more relevant. Fish products consumed in the EU were responsible for the highest amount of food waste and by-products generated outside the EU, equal to more than 5 Mt, due to the large amount of fish imported. On the contrary, meat generated the largest negative contribution, equal to 1.5 Mt, meaning that a fraction of food waste and by-products from meat produced in the EU is associated with the consumption happening in extra-EU countries.

### 3.3 Limitations and future research

This study is affected by several limitations that should be addressed in future research in order to obtain a more comprehensive and robust food waste accounting. These include:

(i) Water leaving and being added to the system was only considered for selected processes (e.g. beer and sugar production). However, as water can make up a large share of the total mass of a waste stream (e.g. in the processing of fruit into fruit concentrate), a complete mapping of water flows would improve the accuracy of the MFA.
(ii) An estimation of the amounts of by-products leaving the FSC was done by assuming that a share of the food waste is recycled, e.g. for animal feed production. These considerations were mainly based on expert judgement, based on Kemna et al. (2017), and may not be fully representative of the EU reality.
(iii) The waste coefficients used to estimate flows of food waste at PP, D&R, and consumption, were based on studies from a limited number of countries and therefore cannot be considered fully representative of the EU.
(iv) The amount of fresh products entering the distribution stage is not provided by any statistical source (as opposed to the manufactured products). Therefore, this quantity was estimated by subtracting from the quantities harvested for human consumption the fresh equivalent of the amounts used by the food manufacturing industry. As the latter was derived by applying conversion coefficients to the manufactured products, which are affected by a degree of uncertainty, the estimate of the fresh products could also be an under/over estimation of reality.
(v) A food waste accounting at EU level entails a significant number of sources of uncertainty that were not considered in this study. We assessed only the uncertainty associated with the waste coefficients used at PP, R&D and consumption and its influence on the results. Nevertheless, the uncertainty associated with other coefficients used in the P&M stage as well as with the input data should be analysed in future research. A possible way to increase the robusteness of the estimation of food waste at EU level would be to perform the accounting exercise at country level using country-specific data (e.g. food waste coefficients for primary production representative for each country), and then to aggregate the data at EU level considering the variations observed.

## 4 Conclusions

This work adopted a high-level top-down approach for food waste accounting at EU level by combining different sources of information, with a breakdown into the major EU food groups at the different stages of the food supply chain. The accounting exercise followed the principles of mass conservation in which input equals output, using closed mass balances through the use of MFA concepts. This work aimed at providing a detailed and comprehensive picture of the EU food supply chain, with a focus on food waste generation, based on MFA and statistical data sources. Such accounting approach is deemed more robust than previous research combining food waste coefficients with statistical data (e.g. Gustavsson et al., 2011), thanks to a more accurate analysis of the flows of food across the FSC obtained through a detailed breakdown of different commodities at processing, retail and distribution, and consumption stages. Furthermore, using a closed mass balance approach can help identifying inconsistencies between datasets, highlighting the need for future work to improve statistical data on food production, trade, and waste generation.

Fruit and vegetables were the food groups presenting the highest amount of food waste overall, with similar amounts generated at the primary production and consumption stages. For most food groups, the highest share of food waste is generated at consumption stage; however, this is not the case for fish, oil crops, and sugar beets. For the former two food groups, most of the waste generation happens at the processing and manufacturing stage, while for the latter at primary production. Distribution and retail is the stage with the lowest share of food waste for all food groups. It is worth to highlight that this study provides an approximate estimate of the level of FW in the EU suitable for prioritisation of various food waste prevention actions. If one intends to use such approach for accurately tracking against targets, coefficients should ideally be replaced by more updated and country/industry specific values.

As pointed out in the previous section, several limitations were identified in this exercise that represent research gaps to be addressed by future research in order to provide a more accurate and comprehensive picture of food waste generation at EU level. This research highlighted the need to increase the amount of primary data on waste generated along the food supply chain. Ultimately, this would allow to robustly capture the food waste generated at EU level, to establish a proper baseline and to accurately track the progress towards SDG target 12.3.
