# Supplementary material for: Quantification of food waste per product group along the food supply chain in the European Union: a mass flow analysis
Source: Resour Conserv Recycl. 2019 Oct;149:479–88. doi: 10.1016/j.resconrec.2019.06.011 (PMC6703187; doi:10.1016/j.resconrec.2019.06.011)
Supplement: Supplementary file 1 [file mmc1.docx]

**Supplementary Information**

**Quantification of food waste per product group and along the food supply chain in the Europe Union: a Mass Flow Analysis**

Carla Caldeira^1^, Valeria De Laurentiis^1^, Sara Corrado^1^, Freija van Holsteijn^2^, Serenella Sala^1,*^

*^1^European Commission-Joint Research Centre, Via Enrico Fermi 2749, I-21027 Ispra (VA), Italy*

*^2^ VHK BV, Rotterdamseweg 386 B-18, 2629 HG, Delft, The Netherlands.* *Present address: Blonk Consultants, Gravin Beatrixstraat 34, 2805 PJ, Gouda, The Netherlands*

* Corresponding author: [serenella](mailto:michela.secchi@ext.ec.europa.eu).sala@ec.europa.eu

Telephone: +39 0332 786417

**Contents**

[1. Primary Production 2](#_Toc10544280)

[2. Processing and manufacturing 3](#_Toc10544281)

[2.1 Meat 3](#_Toc10544282)

[2.2 Fish 5](#_Toc10544283)

[2.3 Dairy and eggs 7](#_Toc10544284)

[2.4 Cereals 7](#_Toc10544285)

[2.5 Fruits, vegetables and potatoes 8](#_Toc10544286)

[2.6 Sugar beets and oil crops 11](#_Toc10544287)

[3. Distribution and retail, and consumption 11](#_Toc10544288)

[4. Mass flow analysis of EU food and waste flows 14](#_Toc10544289)

[5. Comparison with food consumption data 17](#_Toc10544290)

[6. Uncertainty Assessment 18](#_Toc10544291)

[6.1 Quantification of uncertainty factors and coefficient of variation 18](#_Toc10544292)

[6.2 Contribution to variance 25](#_Toc10544293)

[References 26](#_Toc10544294)

## Primary Production

Table S1 summarizes the coefficients (average value) used to calculate the food waste at primary production for each food group obtained from Hartikainen et al. (2018). According to the definitions used by Hartikainen et al. (2018), “side flow percentage” (sf_PP,i_) is the portion of edible and inedible parts of food produced for human consumption not entering the next step of the food supply chain. The authors also provided a “Fusions food waste percentage” (ffw_PP,i_) which is restricting the above definition by excluding food waste used as animal feed, in line with the FUSIONS definition of food waste. In the current work, both coefficients are used to calculate the food waste generated at primary production (FW_pp,i_), as illustrated by equation (2) of the article. Additionally, the amount used as animal feed was calculated following equations S1 and S2.

$\mathrm{SF}_{PP, i}= \frac{P}{1- \mathrm{sf}_{PP,i}}*\mathrm{sf}_{pp,i}$ (S1)

Animal feed = $\mathrm{SF}_{PP, i}- \mathrm{FW}_{PP, i}$ (S2)

Table S1. Coefficients used to calculate food waste in primary production for each food group i obtained from Hartikainen et al. (2018).

| **Food group (i)** | **Side flow percentage (sf_PP,i_) (%)** | **Fusions food waste percentage (ffw_PP,i_) (%)** |
| --- | --- | --- |
| Meat | 0.8 | 0.8 |
| Fish | 0.7 | 0.7 |
| Dairy (milk) | 0.3 | 0.3 |
| Eggs | 3.6 | 3.6 |
| Cereals | 6.5 | 1.4 |
| Fruit | 14.0 | 14.0 |
| Vegetables | 19.2 | 15.8 |
| Potatoes | 10 | 2.5 |
| Sugar beets | 5.0 | 2.5* |
| Oil crops | 3.0 | 3.0** |

*Assumed as potatoes; **Assumed as sf_PP,oil crops_

For each food group, the amount used as animal feed according to the FAO commodity balance sheets (FAO, 2011a), was added to the amount calculated above, to obtain the total flow at primary production used as animal feed (presented in Table 2 of the article). Furthermore, the amount leaving the supply chain at primary production to be used for non-food purposes, was also extracted from the FAO commodity balance sheets (FAO, 2011a), considering what the FAO classify as “other uses” and as “processing” (intended as processing for non-food uses) with the exception of the following groups: fruit, sugar beet, and oil crops, where only “other uses” were considered as the amounts reported as “processing” referred to quantities used for the production of wine, sugar and vegetable oils, included in the Sankey diagram. These amounts are also reported in Table 2 of the article.

## 2. Processing and manufacturing

In this section, we describe how the food waste was estimated in the processing and manufacturing stage for the different food groups.

### 2.1 Meat

The meat produced in the EU expressed in carcass weight (CW) was taken from the FAO commodity balance sheets (FAO, 2011a). Then the net trade (imports minus exports) taken from the FAO trade statistics (FAO, 2011b) was added to the amount produced. Meat food waste and by-products were determined from the slaughtering of the animals and the manufacturing of meat-based products.


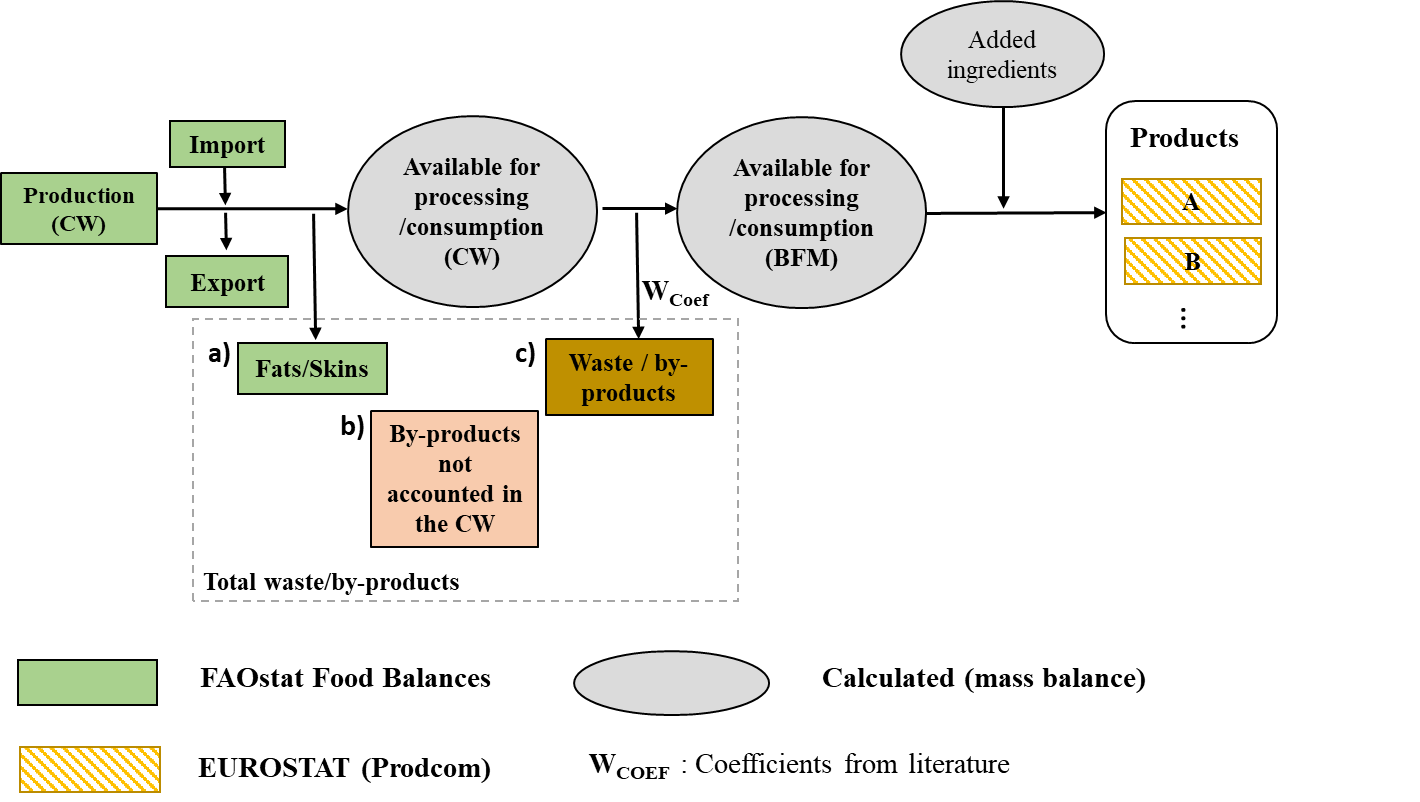


Figure S1: Scheme of the accounting approach adopted for meat commodities at production and processing stage including the main sources of data, CW = carcass weight, BFM = bone free meat

Figure S1 illustrates the process followed to quantify food waste at the processing stage of meat. This is in line with the general procedure illustrated in Figure 1 of the article; however, for this commodity group the modelling included some additional steps. The waste and by-products generated by the production of meat (animal fats, hides and skin and other by-products including bones, blood and inedible organs) were estimated as the sum of three different quantities (identified in Figure S1).

1. Animal fats and hides and skin (by-products) produced at the slaughtering stage as reported by the FAO commodity balance sheets (FAO, 2011a) (green box in Figure S1), equal to 3.4 Mt.
2. By-products generated at slaughtering stage and not included in the carcass weight (CW) (and therefore not accounted for in the commodity balance sheets) (orange box in Figure S1), equal to 11.3 Mt. These by-products were calculated from the total amounts of livestock produced in the EU (expressed in live weight, taken from FAO livestock primary production (FAO, 2011c), multiplied by coefficients calculated from FAOSTAT conversion factors (FAO,2003). The coefficients used are reported in Table S2.
3. Waste and by-products generated from the manufacturing of meat products, calculated as the difference between the meat measured in carcass weight and the bone-free meat (BFM) equivalent that can be obtained from it (brown box in Figure S1). This was calculated using coefficients from the literature that report the ratio between bone free meat and carcass weight, as reported in Table S3. The split between waste and by-products was then done according to Kemna et al. (2017). The resulting values were: 11.4 Mt of by-products and 2.9 Mt of waste.

The sum of all by-products used for non-food purposes resulted in 26.4 Mt, as presented in Table 2 of the article.

Table S2 Coefficients used to determine the waste and by-products not included in the carcass weight calculated from FAO (2003).

|  | **Bovine** | **Sheep** | **Pigs** | **Chickens** |
| --- | --- | --- | --- | --- |
| **Waste and byproducts / live weight** | 32% | 31% | 9% | 22% |

Table S3. Coefficients used to calculate the amount bone free meat available for each specie.

| **Animal** | **Bone free meat/Carcass weight** | **Source** |
| --- | --- | --- |
| **Bovine** | 69% | Clune et al. (2017) |
| **Sheep** | 66% | Clune et al. (2017) |
| **Pigs** | 59% | Sonesson et al. (2010) |
| **Chickens** | 77% | Sonesson et al. (2010) |

The amount of meat available for processing and consumption expressed in bone-free meat was then calculated through a mass balance (Figure S1). To conclude, this quantity was compared with the total amount of meat-based products as reported in Prodcom (EUROSTAT, 2011), to calculate the ingredients added at processing stage by performing a mass balance.

### 2.2 Fish

The amount of fish available in the EU for processing and fresh consumption was calculated by adding to the fish produced in EU, taken from the FAO commodity balance sheets (FAO, 2011a), the net trade (imports minus exports), taken from EUMOFA (2014), and subtracting the amount of fish used for animal feed and other (non-food) uses as reported in commodity balance (FAO, 2011a, 2011b). The amount of processed fish products was taken from Prodcom (EUROSTAT, 2011) and for each type of product, coefficients (table S4) from the literature were applied to calculate the “fresh fish equivalent”. For example, for the product “Smoked Pacific, Atlantic and Danube salmon (including fillets)”, the quantity produced in Europe according to Prodcom is: 0.129 Mt. This quantity is multiplied by a coefficient of 1.92 (for smoked salmon, Table S4). The result, equal to 0.247 Mt, is the fresh fish equivalent necessary to produce the quantity of smoked salmon reported.

The fish consumed fresh was calculated by subtracting from the total supply of fish the total amount of fish necessary to produce the processed products (the sum of all the “fresh fish equivalents”), as illustrated by Figure 1 of the article.

The processing by-products and waste were calculated in two alternative ways, according to the type of products.

- For products where no added ingredients and no evaporation of water is assumed, by-products and waste were calculated as the difference between fresh equivalent and product.
- In all the other cases (e.g. smoked cod), the by-products and waste were calculated as the inedible fraction of the fresh equivalent. A mass balance was then applied to calculate the amount of evaporated water and/or added ingredients.

The coefficients used to determine the inedible fraction of different fish species are presented in table S5. In the case of the smoked salmon reported above, the processing waste and by-products is calculated by multiplying the fresh fish equivalent (1.92 Mt) by a coefficient of 0.36 (inedible fraction of salmon, Table S5), resulting in a value of 0.089 Mt of waste and by products. Finally, the water evaporated is calculated as the difference between the weight of fresh fish equivalent and the sum of the product weight and the weight of waste and by products, resulting in a value of 0.029 Mt. This was repeated for each fish product extracted from Prodcom, resulting in a total processing waste of 4.6 Mt. Of this amount, it was assumed that 1.5 Mt would be used for the production of animal feed (and therefore considered a by-product), based on Jackson and Newton (2016), as presented in Table 2 of the article, and the remaining 3.1 Mt would be wasted.

Table S4. Coefficients used to calculate the fresh fish equivalent in each product obtained from FAO (1990)

| **Product** | **Fresh fish equivalent/product weight** |
| --- | --- |
| Fillet, demersal | 3.07 |
| Fillet, pelagic | 2.17 |
| Fillet, freshwater | 3.55 |
| Any fish, Fillet | 2.93 |
| Mince, demersal | 3.21 |
| Mince, pelagic | 2.17 |
| Mince, freshwater | 3.55 |
| Any fish, Mince | 2.98 |
| Any fish, Smoked | 1.61 |
| Smoked salmon | 1.92 |
| Smoked herring | 1.49 |
| Salmon wet or in brine | 1.50 |
| Herrings wet or in brine | 1.39 |
| Sardines prepared or preserved | 1.07 |
| Tuna, preserved, canned | 1.44 |
| Mackerel, preserved | 1.46 |
| Anchovies, preserved | 1.63 |
| Other demersal, preserved | 1.60 |
| Other pelagic, preserved | 1.39 |
| Any fish, preserved | 1.50 |
| Crustaceans, meat | 4.22 |
| Mussels, meat | 4.07 |

Table S5 Coefficients to calculate the inedible fraction of different fish species obtained from Waterman (2001)

| **Fresh product** | **Inedible fraction/fresh weight** |
| --- | --- |
| Salmon | 0.36 |
| Herring | 0.47 |
| White fish average | 0.57 |
| Sardine | 0.30 |
| Tuna | 0.48 |
| Makerel | 0.49 |
| Anchovies | 0.54 |

### 2.3 Dairy and eggs

The milk available for processing in the EU was obtained by subtracting from the total milk available in the EU (production + stock variation + import – export) the amount used on farms and for non-food purposes, as reported in the commodity balance sheets (FAO, 2011a). The food waste produced at the processing stage for dairy products was calculated according to Kemna et al. (2017). According to this source, the only by-product leaving the human food supply chain is a fraction of the whey produced during cheese-making, used for the production of animal feed and equal to 11.4 Mt.

Of the total eggs available at EU level, taken from the commodity balance sheet (FAO, 2011a), it was assumed that 65% would be distributed fresh and 35% would be processed in products such as liquid, dried and frozen egg products (Agra CEAS Consulting, 2008). The processing stage led to the generation of by-products (0.3 Mt of eggshells used as fertilizers), calculated according to Rimestad et al. (2000), and 0.1 Mt of waste, calculated using a coefficient of 5% according to (Roland 1988).

### 2.4 Cereals

The amount of cereals available in EU for processing was calculated by subtracting from the cereals available in EU (production + stock variation +import – export) the amount of cereals used for animal feed, seeds production and other (non-food) uses as reported in commodity balance sheets (FAO, 2011d). For this purpose, the following commodities were considered: common wheat, barley, rye, maize, oats, rice and other cereals.

The processing and manufacturing stage was divided in two sub-stages: milling and the production of cereal-based products. The waste and by-products from milling were calculated as the difference between the cereal available for processing and the amount of the intermediate cereal-based products produced in EU, reported in Prodcom, and categorised as follow: rice, flour, starch (excluding potatoes starch), malt, and other intermediate cereal-based products, e.g. dough. This amount, equal to 20.7 Mt, was assumed to be a by-product all used for animal feed production.

The amount of waste and by-products from the production of cereal-based products was calculated as the amount of cereal-based products available for consumption in EU, calculated from Prodcom data (EUROSTAT, 2011) considering the domestic production and the trade balance, minus the amount of milled cereal in final products and the amount of flour for direct consumption.

The amount of milled cereal in final products was calculated by multiplying data on production of cereal-based products and the coefficients reported in Table S6, which express the ratio between the weight of milled cereals in the final cereal-based products and the weight of the cereal-based products.

The amount of flour for direct consumption was calculated as the difference between the flour available in EU (production + import – export, as from Prodcom) and the flour used in final products, e.g. bread.

The resulting amount of waste and by-products was equal to 7.4 Mt, of which 4.9 Mt were assumed to be used as animal feed and 2.5 to be wasted, following the split by Kemna et al., (2017). Therefore, the total by-products from the manufacturing of cereals used as animal feed were equal to 25.6 Mt (as presented in Table 2 of the article).

Table S6. Coefficients calculated as the weight of milled cereals in the final cereal-based products divided by the weight of the cereal-based products

| **Product** | **Coefficient** | **Source** |
| --- | --- | --- |
| Beer | 0.15 | Own calculations* |
| Bread | 0.75 | Kemna et al. (2017) |
| Pastry | 0.25 |  |
| Biscuits | 0.50 |  |
| Pasta | 1.00 |  |

* *Calculated as 1-(available malt/produced beer), data from Prodcom*

### 2.5 Fruits, vegetables and potatoes

The amounts of fruit, vegetables and potatoes available in EU for fresh consumption and processing were calculated by subtracting from the amounts available in EU (production + stock variation + import – export) the amounts used for animal feed, other (non-food) uses and seeds production as reported in commodity balance sheets (FAO, 2011b, 2011d).

Then the supply of processed fruit/vegetable/potato products was taken from Prodcom (EUROSTAT, 2011) and for each type of product coefficients from the literature (Table S7) were applied to calculate the “fresh equivalent”.

Table S7. Coefficients used to calculate the “fresh equivalent” of each product.

| **Product** | **Fresh equivalent / product weight** | **Source** |
| --- | --- | --- |
| Citrus juice | 2.00 | FAO (2003) |
| Grapefruit juice | 2.00 |  |
| Pineapple juice | 2.50 |  |
| Grape juice (including grape must) | 1.43 |  |
| Apple juice | 1.43 |  |
| Other juice | 1.43 |  |
| Citrus jams and jellies | 0.59 | Herbstreith & Fox (2018) |
| Other jams | 0.50 |  |
| Dried grapes | 5.88 | FAO (2003) |
| Dried fruits, other | 2.67 |  |
| Frozen fruits and nuts | 1.21 |  |
| Other fruit preparations | 1.21 |  |
| Wine | 1.41 |  |
| Frozen vegetables | 1.16 |  |
| Dried onions | 11.04 |  |
| Dried mushrooms | 12.50 | Calculations performed in this study (based on water content or on recipes) |
| Dried vegetables | 11.04 | FAO (2003) |
| Canned beans | 0.29 | Calculations performed in this study (based on water content or on recipes) |
| Canned peas | 0.26 |  |
| Canned tomatoes | 1.20 | Rickard and Sumner (2006) |
| Un-concentrated tomato paste | 1.16 | FAO (2003) |
| Concentrated tomato paste | 6.25 |  |
| Preserved mushrooms | 0.91 |  |
| Preserved sauerkraut | 2.00 | Calculations performed in this study (based on water content or on recipes) |
| Preserved asparagus | 1.59 | FAO (2003) |
| Preserved sweetcorn | 2.22 |  |
| Other preserved vegetables | 1.59 |  |
| Frozen potatoes, uncooked or cooked by steaming or boiling in water | 2.17 | Marwaha et al. (2010) |
| Frozen potatoes, prepared or preserved (including potatoes cooked or partly cooked in oil and then frozen; excluding by vinegar or acetic acid) | 2.17 |  |
| Dried potatoes whether or not cut or sliced but not further prepared | 5.81 |  |
| Dried potatoes in the form of flour, meal, flakes, granules and pellets | 5.21 |  |
| Potatoes prepared or preserved in the form of flour, meal or flakes (excluding frozen, crisps, by vinegar or acetic acid) | 5.21 |  |
| Potatoes prepared or preserved, including crisps (excluding frozen, dried, by vinegar or acetic acid, in the form of flour, meal or flakes) | 3.45 |  |
| Potato starch | 5.88 | FAO (2003) |

At this point the amount of fruit/vegetables/potatoes consumed fresh was calculated by subtracting from the total supply the total amount of fresh products necessary to produce the processed products (the sum of all the “fresh equivalents”) (as illustrated in Figure 1 of the article).

Then the by-products and processing waste were calculated in two alternative ways, according to the type of product considered.

- For products where no added ingredients and no evaporation of water is assumed, by-products and waste were calculated as the difference between fresh equivalent and product.
- In all the other cases (e.g. dried vegetables, jams), the by-products and waste were calculated as the inedible fraction of the fresh equivalent. A mass balance was then applied to calculate the amount of evaporated water and/or added ingredients.

The coefficients calculating the inedible fraction of different products were taken from De Laurentiis et al. (2018) for fruit and vegetables and Somsen (2004) for potatoes.

Table S8 Coefficients used to calculate the inedible fraction of fruit and vegetables (De Laurentiis et al. 2018) and potatoes (Somsen 2004).

| **Processed product** | **Fresh product assumed** | **Inedible fraction/fresh weight** |
| --- | --- | --- |
| Citrus jams and jellies | Orange | 0.24 |
| Other jams | Strawberries | 0.05 |
| Dried onions | Onions | 0.11 |
| Dried/preserved mushrooms | Mushrooms | 0.11 |
| Canned beans | Beans | 0.66 |
| Canned peas | Peas | 0.66 |
| Canned tomatoes/tomato paste | Tomatoes | 0.13* |
| Preserved sauerkraut | Cabbage | 0.20 |
| Preserved asparagus | Asparagus | 0.45 |
| Preserved sweetcorn | Sweetcorn | 0.44 |
| Frozen/dried/preserved potatoes | Potatoes | 0.08 |

*the value for tomatoes was taken from (Leoni, 1997)

Of the resulting amounts obtained, a split between waste and by-product was done following the approach adopted by Kemna et al. (2017), leading to a total of 10.1 Mt of by-products used for animal feed and 10.8 Mt of waste generated from the processing of fruit, vegetables and potatoes.

### 2.6 Sugar beets and oil crops

The sugar beet available for processing is taken from the commodity balance sheet (FAO, 2011d) (under “Sugar beet”). The water released when it is refined into sugar is calculated considering a water content of 78% (Kemna et al., 2017). The refined sugar produced in the EU is taken from the commodity balance sheet (under “refined sugar”). A mass balance was performed to calculate the processing waste and by-products, resulting in 8.5 Mt. This quantity was assumed to be used for the production of animal feed.

The oil crops supply was taken from the commodity balance sheets (considering both “processing” and “food supply” entries), and considering the following crops: olives, rapeseed, soybeans, sunflower seeds and cottonseeds. For all crops apart from olives, it was assumed that 46% would leave the food supply chain (FEDIOL, 2013). Then the supply of products (i.e. preserved olives, olive oil, rapeseed oil, soybean oil, sunflower oil and cottonseed oil) was taken from Prodcom (EUROSTAT, 2011). In the case of olives, a mass balance between the amount available for processing and the final product (olive oil) was conducted to calculate the amount of waste (waste pomace and wastewater), resulting in 10 Mt (Table 1 of the article). For the remaining crops, a mass balance was conducted between the amounts available for processing and the related products, resulting in 14.7 Mt of oil cake, assumed to be used for the production of animal feed (as presented in Table 2 of the article).

## 3. Distribution and retail, and consumption

The coefficients used to calculate the amount of food waste generated at retail and distribution and consumption level are reported in table S9. These coefficients are taken from a review of scientific and grey literature on food waste, which builds on the review conducted by Xue et al. (2017). The studies presented in table S9 were selected from the ones included in the review if they:

- assessed food waste produced either in the EU28 or in a country belonging to the EU28;
- provided waste coefficients calculated as the ratio between the food waste generated at a step of the supply chain and the amount of food entering that step of the supply chain;
- used either primary data or a combination of modelling and statistical data (in other words studies only based on literature were excluded).

For some commodities and food supply chain stages (e.g. waste of chicken at retail stage) more than one study met the selection criteria. In these cases, a quality judgement was done to choose the study that either seemed more robust (e.g. giving preference to studies that collected primary data using significant sample sizes, e.g. WRAP 2014) or more representative of the EU28 (e.g. De Laurentiis et al. 2018). When no waste coefficient was available from the literature, the coefficient of a similar product was used as a proxy. Additionally, Table S10 presents the coefficients used to calculate the avoidable food waste generated by households and food services. For all the products that only appear in Table S9, it is assumed that all the waste generated is avoidable (as these products are 100% edible once they reach the consumption stage, e.g. processed fruit products or rice), and therefore no additional coefficients were necessary to calculate the avoidable component.

Table S9. Coefficients used to calculate the amount of food waste generated at distribution and retail, and at consumption level (for households and food services). All coefficients represent the share of the flow entering the stage of the FSC that is wasted.

| **Product** | **Distribution and retail waste** | | **Household waste** | | **Food service waste** | |
| --- | --- | --- | --- | --- | --- | --- |
|  | % | source | % | source | % | source |
| **Beef** | 3.9% | Mena et al. (2014) | 13.0% | WRAP (2014) | 17.4% | Beretta et al. (2013) |
| **Pork** | 3.8% |  | 18.0% |  | 18.3% |  |
| **Poultry** | 3.7% |  | 34.0% |  | 50.3% |  |
| **Lamb** | 3.7% |  | 12.0% |  | 17.4% |  |
| **Fish** | 3.6% | Beretta et al. (2013) | 12.0% |  | 22.3% |  |
| **Milk** | 0.8% |  | 7.0% |  | 5.1% |  |
| **Cheese** | 0.8% |  | 9.0% |  | 12.1% |  |
| **Yogurt** | 0.8% | Assumed from the coefficient of milk | 9.0% |  | 5.1% | Assumed from the coefficient of milk |
| **Butter** | 0.3% | Beretta et al. (2013) | 8.0% | Beretta et al. (2013) | 4.7% | Beretta et al. (2013) |
| **Eggs** | 1.4% |  | 23.0% | WRAP (2014) | 36.0% |  |
| **Rice** | 0.3% |  | 6.0% |  | 18.0% |  |
| **Household flour** | 0.5% | assumed from the coefficient of pasta | 6.0% | assumed from the coefficient of pasta | 6.0% | assumed from the coefficient of household waste |
| **Bread** | 4.8% | Beretta et al. (2013) | 29.0% | (WRAP, 2014) | 20.0% | Beretta et al. (2013) |
| **Pastry** | 4.8% |  | 15.0% |  | 20.0% |  |
| **Biscuits** | 0.5% | assumed from the coefficient of pasta | 4.0% |  | 4.0% | assumed from the coefficient of household waste |
| **Pasta** | 0.5% | Beretta et al. (2013) | 6.0% |  | 18.0% | Beretta et al. (2013) |
| **Beer** | 0.5% | assumed from the coefficient of pasta | 5.5% |  | 5.5% | assumed from the coefficient of household waste |
| **Breakfast cereals** | 0.5% | assumed from the coefficient of pasta | 10.0% |  | 10.0% | assumed from the coefficient of household waste |
| **Fresh fruits** | 2.7% | WRAP (2011) | 29.7% | De Laurentiis et al. (2018) | 28.2% | Beretta et al. (2013) |
| **Processed fruits** | 0.4% | Beretta et al. (2013) | 11.0% | WRAP (2009) | 10.9% |  |
| **Fresh vegetables** | 1.9% | WRAP (2011) | 31.0% | De Laurentiis et al. (2018) | 31.1% |  |
| **Processed vegetables** | 0.2% | Beretta et al. (2013) | 12.0% | WRAP (2009) | 9.3% |  |
| **Fresh potatoes** | 2.0% | Willersinn et al. (2015) | 30.3% | De Laurentiis et al. (2018) | 30.6% |  |
| **Processed potatoes** | 0.0% |  | 13.0% | WRAP (2014) | 9.3% |  |
| **Refined sugar** | 3.3% | Beretta et al. (2013) | 12.0% | Beretta et al. (2013) | 13.3% |  |
| **Vegetable oils** | 0.8% |  | 16.0% | WRAP (2009) | 18.5% |  |
| **Olives (preserved)** | 0.4% | assumed from the coefficient of processed fruit | 11.0% | assumed from the coefficient of processed fruit | 10.9% | assumed from the coefficient of processed fruit |
| **Tree nuts** | 0.4% | assumed from the coefficient of processed fruit | 11.0% | assumed from the coefficient of processed fruit | 10.9% | assumed from the coefficient of processed fruit |

Table S10. Coefficients used to calculate the amount ofedible food waste generated at household and food service stage. All coefficients represent the share of the flow entering the stage of the FSC that is wasted.

| **Product** | **Household edible waste** | | **Food service edible waste** | |
| --- | --- | --- | --- | --- |
|  | % | source | % | source |
| **Beef** | 8.0% | WRAP (2014) | 9.4% | Beretta et al. (2013) |
| **Pork** | 13.0% |  | 10.3% |  |
| **Poultry** | 13.0% |  | 10.1% |  |
| **Lamb** | 5.0% |  | 9.4% |  |
| **Fish** | 10.0% |  | 8.5% |  |
| **Eggs** | 6.0% |  | 7.2% |  |
| **Fresh fruits** | 10.5% | De Laurentiis et al. (2018) | 14.0% |  |
| **Fresh vegetables** | 12.3% |  | 16.6% |  |
| **Fresh potatoes** | 12.3% |  | 16.1% |  |

## 4. Mass flow analysis of EU food and waste flows

The following tables report the results of the mass flow analysis of EU food and waste flows performed, respectively for plant-based products (Table S11) and for animal-based products (Table S12). The values reported were used to build the Sankey diagram (Figure 2 of the manuscript).

Table S11. Mass flow analysis of plant-based products across the food supply chain.

| **Stage** | **Flow description** | **Amounts per food group [Mt]** | | | | | |
| --- | --- | --- | --- | --- | --- | --- | --- |
|  |  | **Sugarbeet** | **Oilcrops** | **Potatoes** | **Vegetables** | **Fruit** | **Cereals** |
| **Primary Production** | Total yield | 131.7 | 44.4 | 69.3 | 88.8 | 72.1 | 314.6 |
|  | Side flow* | 6.2 | 0.9 | 4.8 | 16.3 | 11.1 | 5.4 |
|  | o/w animal feed | 3.1 | 0.0 | 3.6 | 2.9 | 0.0 | 4.3 |
|  | o/w Food Waste | 3.1 | 0.9 | 1.2 | 13.4 | 11.1 | 1.2 |
|  | Import + stock | 0.6 | 14.8 | -1.1 | 4.3 | 9.5 | 11.9 |
|  | Production | 125.1 | 43.0 | 62.4 | 71.7 | 62.0 | 294.2 |
|  | Seeds |  | 0.3 | 4.7 | 0.3 |  | 10.0 |
|  | Export | 0.0 | 1.0 | 1.7 | 2.7 | 3.3 | 24.2 |
|  | Non-food | 6.8 | 18.9 | 6.7 | 0.1 | 0.1 | 30.7 |
|  | Animal feed | 0.2 | 2.3 | 5.5 | 4.4 | 0.2 | 163.1 |
|  | Amounts leaving primary production (going to processing) | 118.7 | 35.4 | 26.1 | 24.8 | 43.4 | 78.2 |
|  | Amounts leaving primary production (fresh for distribution) |  |  | 16.7 | 43.7 | 24.5 |  |
| **Processing and Manufacturing** | Added ingredients |  |  |  | 1.7 | 0.9 | 13.0 |
|  | By-products to animal feed | 8.5 | 14.6 |  | 2.6 | 7.5 | 25.6 |
|  | Food Waste |  | 10.0 | 2.1 | 2.6 | 6.1 | 2.5 |
|  | Water leaving the supply chain | 92.6 |  | 15.9 | 6.1 | 0.5 | -32.7 |
|  | Processed food products leaving P&M | 17.6 | 10.7 | 8.2 | 15.1 | 30.2 | 93.4 |
| **Distribution and Retail** | Import + stock of processed food products | -2.0 | 1.3 | 0.0 | 1.0 | 4.9 | 2.1 |
|  | Export of processed food products | 1.4 | 1.3 | 1.2 | 1.2 | 3.2 | 4.2 |
|  | Processed products going to non- food | 1.0 |  | 0.5 |  |  |  |
|  | Food waste | 0.4 | 0.1 | 0.3 | 0.9 | 0.8 | 1.7 |
|  | Amounts of fresh and processed going to consumption | 12.8 | 10.7 | 22.8 | 57.7 | 55.7 | 89.6 |
| **Consumption** | Entering Household | 10.9 | 9.1 | 19.4 | 49.1 | 47.3 | 64.2 |
|  | Household food waste | 1.3 | 1.4 | 4.9 | 12.2 | 8.6 | 8.0 |
|  | Consumed at household | 9.6 | 7.7 | 14.5 | 36.9 | 38.7 | 56.2 |
|  | Entering Food service | 1.9 | 1.6 | 3.4 | 8.7 | 8.4 | 25.3 |
|  | Food service food waste | 0.3 | 0.3 | 0.8 | 2.2 | 1.5 | 2.2 |
|  | Consumed at food service | 1.7 | 1.3 | 2.6 | 6.5 | 6.8 | 23.1 |

*the side flow is calculated only for the portion of crops harvested for food production

Table S12. Mass flow analysis of animal-based products across the food supply chain.

| **Stage** | **Flow description** | **Amounts per food group [Mt]** | | | |
| --- | --- | --- | --- | --- | --- |
|  |  | **Meat** | **Fish** | **Dairy** | **Eggs** |
| **Primary Production** | Total yield | 66.6 | 6.6 | 156.0 | 7.1 |
|  | Side flow/Food waste* | 0.5 | 0.0 | 0.5 | 0.3 |
|  | Import + stock | 0.9 | 5.2 | -0.1 | 0.0 |
|  | Production | 66.0 | 6.6 | 155.5 | 6.9 |
|  | Export | 5.3 | 1.5 | 0.3 | 0.1 |
|  | Non-food |  | 0.3 | 0.4 | 0.6 |
|  | Animal feed |  | 1.8 | 4.6 |  |
|  | Amounts leaving primary production (going to processing) | 61.7 | 8.1 | 150.2 | 2.2 |
|  | Amounts leaving primary production (fresh for distribution) |  | 0.2 |  | 4.0 |
| **Processing and Manufacturing** | Added ingredients | 17.7 | 0.4 |  |  |
|  | By-products to animal feed |  | 1.5 | 11.4 |  |
|  | By products to non-food | 26.4 |  |  | 0.3 |
|  | Food Waste | 2.9 | 3.1 | 1.1 | 0.1 |
|  | Water leaving the supply chain |  | 0.1 | 74.2 | 0.3 |
|  | Processed food products leaving P&M | 50.1 | 3.8 | 63.5 | 1.5 |
| **Distribution and Retail** | Import + stock of processed food products | 0.7 | 3.3 | 0.3 | 0.0 |
|  | Export of processed food products | 4.5 | 1.2 | 3.2 | 0.0 |
|  | Food Waste | 1.7 | 0.2 | 0.4 | 0.1 |
|  | Amounts of fresh and processed going to consumption | 44.6 | 5.9 | 60.2 | 5.4 |
| **Consumption** | Entering Household | 37.9 | 4.4 | 51.1 | 4.6 |
|  | Household food waste | 7.3 | 0.5 | 4.2 | 1.1 |
|  | Consumed at household | 30.6 | 3.8 | 46.9 | 3.6 |
|  | Entering Food service | 6.7 | 1.5 | 9.0 | 0.8 |
|  | Food service food waste | 1.7 | 0.3 | 0.6 | 0.3 |
|  | Consumed at food service | 5.0 | 1.2 | 8.4 | 0.5 |

* for animal based products the food waste represents 100% of the side flow.

Figure S2 presents similar information to Figure 3 of the manuscript. However, we believe it is better highlighting how, the relative contribution of the different stages of the FSC to the total food waste generated, varies significantly across food groups.


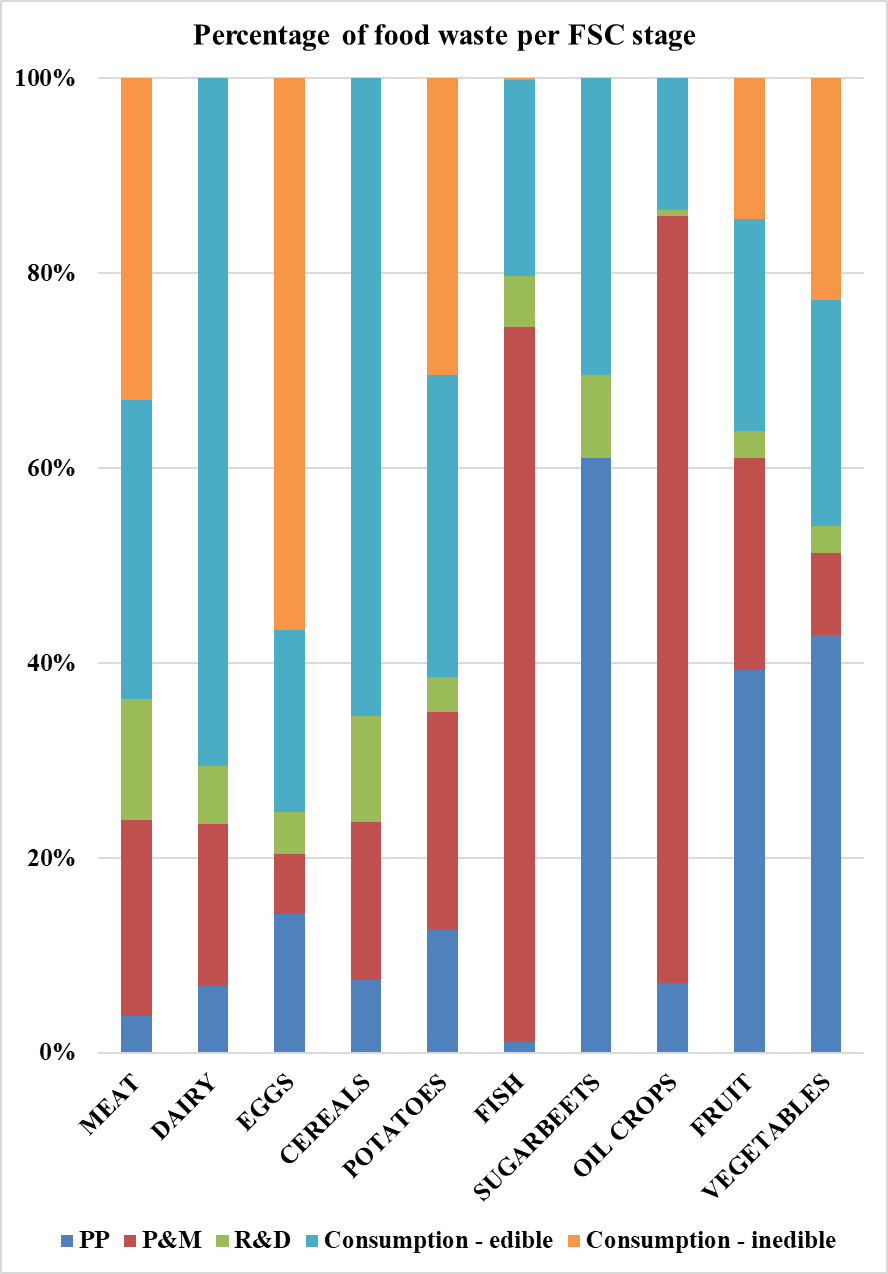


Figure S2: Contribution of the stages of the FSC to the total food waste generated for each food group. PP: primary production, P&M: processing and manufacturing, R&D: retail and distribution.

## 5. Comparison with food consumption data

The mass balance exercise ends at the consumption stage having as one of the outputs the amount of food consumed. To check these values, this amount was compared with the average food consumption data reported in EEA (2017). The results are presented in table S13. This table also reports, in the third column, the deviation of the values calculated in this study from the ones reported by the EEA; a positive deviation value means that we obtained a higher value of consumption for that specific food group compared to the one obtained by the EEA, while negative deviation values mean the opposite.

Table S13 Average food consumption data obtained in this study through mass balance and EEA figures (EEA, 2017).

| **Food group** | **Food consumption**  **(kg / person /day)** | | **Deviation (%) relatively to the EEA study**  **(A-B)/B** |
| --- | --- | --- | --- |
|  | **This study (A)** | **EEA (B)** |  |
| **Meat** | 0.194 | 0.175 | 11% |
| **Fish** | 0.027 | 0.027 | 1% |
| **Dairy** | 0.301 | 0.266 | 13% |
| **Eggs** | 0.022 | 0.019 | 17% |
| **Cereals** | 0.432 | 0.277 | 56% |
| **Fruit** | 0.117 | 0.167 | -30% |
| **Vegetables** | 0.236 | 0.210 | 12% |
| **Potatoes** | 0.093 | 0.118 | -21% |
| **Sugar** | 0.061 | 0.036 | 70% |
| **Vegetable oils** | 0.049 | 0.033 | 49% |
| **Total** | **1.534** | **1.328** | **15%** |

## 6. Uncertainty Assessment

### 6.1 Quantification of uncertainty factors and coefficient of variation

Table S14 (modified from Beretta et al. 2013) illustrates the criteria followed in assigning the pedigree scores to assess the quality of the data sources used. A pedigree score was attributed to each data source considering the following indicators: reliability, completeness, temporal, geographical, further technological correlation and sample size. Additionally, the indicator ‘basic uncertainty’ is considered to account for intrinsic variability and stochastic error of the coefficients. The uncertainty factors attribute to each score for each indicator is presented in table S15.

The pedigree scores attributed to waste coefficients used in this study are presented in Table S16, including the uncertainty factor. The uncertainty factor was calculated according to Equation S1. Then, according to Muller et al. (2014), the coefficient of variation (CV, %) was calculated using Equation S2 and the upper and lower values for the uniform distribution using Equations S3. The CV of each waste coefficient, the upper and lower values considered for the uniform distribution, and the variation of the extremes relatively to the coefficient value are presented in Table S17 for primary production, Table S18 for retail, Table S19 for households and Table S20 for food service.

Table S14: Pedigree matrix (modified from Beretta et al. 2013). Verified data refers to data published in official statistics and public documents or to data verified by on-site check, cross check or mass/energy balance. Sample size refers to the number of data entries that originated the waste coefficient (e.g. number of households where surveys were conducted. Technological correlation considers, if data derives from the enterprises and the processes which cause the corresponding food waste and if it refers to the food group (material) assessed. The market considered for the assessment of completeness, in this analysis, refers to Europe.

| **Pedigree score** | **1** | **2** | **3** | **4** | **5 (default)** |
| --- | --- | --- | --- | --- | --- |
| **Reliability** | Verified data based on measurements | Verified data partly based on assumptions or non-verified data based on measurements | Non-verified data partly based on qualified estimates | Qualified estimate (e.g. by industrial expert); data derived from theoretical information | Non-qualified estimate |
| **Temporal correlation** | 2015-2018 | 2012-2014 | 2008-2012 | 2005-2007 | before 2005 |
| **Geographical correlation** | Data from area under study | Average data from larger area in which the area under study is included, or from smaller area included in the area under study | Data from area with similar production conditions | Data from area with roughly similar production conditions | Data from unknown or distinctly different area (North America instead of Middle East, OECD-Europe instead of Russia) |
| **Further technological correlation** | Origin of data: - enterprises - processes - materials -> all 3 of them correspond to study | Origin of data: - enterprises - processes - materials -> 2 of them correspond to study | Origin of data: - enterprises - processes - materials -> 1 of them corresponds to study | Origin of data: - enterprises - processes - materials -> 1 of them related to study | Data from different technology |
| **Completeness** | Representative data from all sites relevant to the market considered, over an adequate period to even out normal fluctuations | Representative data from >50% of the sites relevant to the market considered, over an adequate period to even out normal fluctuations | Representative data from only some sites (<<50%) relevant to the market considered or >50% of sites but for shorter periods | Representative data from only one site relevant to the market considered or several sites but for shorter periods | Representativeness unknown or data from a small number of sites and for shorter periods |
| **Sample size** | > 100 | > 20 | > 10 | > 2 | Unknown |

**Table S15**: Uncertainty factors, applied with the pedigree matrix (from Beretta et al. 2013)

|  | Pedigree score | | | | |
| --- | --- | --- | --- | --- | --- |
| Indicator | 1 | 2 | 3 | 4 | 5 |
| U1 Reliability | 1 | 1.05 | 1.10 | 1.20 | 1.5 |
| U2 Temporal correlation | 1 | 1.03 | 1.10 | 1.20 | 1.5 |
| U3 Geographical correlation | 1 | 1.01 | 1.02 | 1.05 | 1.1 |
| U4 Further technological correlation | 1 | 1.10 | 1.20 | 1.50 | 2.0 |
| U5 Completeness | 1 | 1.02 | 1.05 | 1.10 | 1.2 |
| U6 Sample size | 1 | 1.02 | 1.05 | 1.10 | 1.2 |
| U7 basic uncertainty factor | 1.05 | | | | |

**Equation S1**

$$GSD= {exp}^{\sqrt{\left[ \ln\left( U1 \right) \right]^{2}+\left[ \ln\left( U2 \right) \right]^{2}+\left[ \ln\left( U3 \right) \right]^{2}+\left[ \ln\left( U4 \right) \right]^{2}+\left[ \ln\left( U5 \right) \right]^{2}+\left[ \ln\left( U6 \right) \right]^{2}+\left[ \ln\left( Ub \right) \right]^{2}}}$$

Were:

U1: uncertainty factor of reliability

U2: uncertainty factor of temporal correlation

U3: uncertainty factor of geographical correlation

U4: uncertainty factor of further technological correlation

U5: uncertainty factor of completeness

U6: uncertainty factor of sample size

Ub: basic uncertainty factor

**Equation S2**

$$CV=\sqrt{exp({ln}^{2} \sigma_{g})-1}$$

Were $\sigma_{g}$ is the geometric standard deviation (GSD).

**Equation S3**

$$CV= \frac{b-a}{\sqrt{3}(a+b)}$$

$$\mu=\frac{a+b}{2}$$

Were a and b are the upper and lower limit of the uniform distribution and $\mu$ the waste coefficient average value.

Table S16: Pedigree scores and uncertainty estimation for the waste coefficients obtained from the different studies.

| **Reference** | **Reliability** | **Completeness** | **Temporal correlation** | **Geographical correlation** | **Technological correlation** | **sample size** | **Uncertainty Factor** |
| --- | --- | --- | --- | --- | --- | --- | --- |
| WRAP (2014) | 1 | 4 | 3 | 2 | 1 | 1 | 1.15 |
| Mena (2014) | 2 | 4 | 3 | 2 | 2 | 3 | 1.20 |
| Hartikenen (2017) | 2 | 4 | 2 | 2 | 2 | 1 | 1.17 |
| WRAP (2009) | 2 | 3 | 4 | 2 | 1 | 1 | 1.22 |
| WRAP (2011) | 2 | 3 | 3 | 2 | 1 | 2 | 1.14 |
| De Laurentiis (2018) | 2 | 3 | 3 | 2 | 1 | 1 | 1.14 |
| Willersinn et al. (2015) - | 2 | 4 | 2 | 2 | 1 | 2 | 1.13 |
| Beretta (2013)  food service | 3 | 4 | 3 | 3 | 4 | 1 | 1.55 |
| Beretta (2013)  retail | 2 | 3 | 3 | 3 | 2 | 5 | 1.27 |

Table S17: Uncertainty factors, coefficient of variation (CV, %), lower and upper values for the waste coefficient and the variation of the limits relatively to the value of the coefficient for the food waste (ffw) and side flow (Sf) coefficients, used to determine food waste at primary production

| Coefficient | Food group | **Coeff** | **Unc. Factor** | **CV** | **Coeff. Lower value** | **Coeff. Upper value** | **Variation** |
| --- | --- | --- | --- | --- | --- | --- | --- |
| **Food waste (ffw)** | **Meat** | 0.8% | 1.17 | 16% | 0.6% | 1.0% | 27% |
|  | **Fish** | 0.7% | 1.17 | 16% | 0.5% | 0.9% | 27% |
|  | **Dairy** | 0.3% | 1.17 | 16% | 0.2% | 0.4% | 27% |
|  | **Eggs** | 3.6% | 1.17 | 16% | 2.6% | 4.6% | 27% |
|  | **Cereals** | 1.4% | 1.17 | 16% | 1.0% | 1.8% | 27% |
|  | **Fruits** | 14.0% | 1.17 | 16% | 10.2% | 17.8% | 27% |
|  | **Vegetables** | 15.8% | 1.17 | 16% | 11.5% | 20.1% | 27% |
|  | **Potatoes** | 2.5% | 1.17 | 16% | 1.8% | 3.2% | 27% |
|  | **Sugarbeets** | 2.5% | 1.17 | 16% | 1.8% | 3.2% | 27% |
|  | **Oil crops** | 3.0% | 1.17 | 16% | 2.2% | 3.8% | 27% |
| **Side flow (Sf)** | **Meat** | 0.8% | 1.17 | 16% | 0.6% | 1.0% | 27% |
|  | **Fish** | 0.7% | 1.17 | 16% | 0.5% | 0.9% | 27% |
|  | **Dairy** | 0.3% | 1.17 | 16% | 0.2% | 0.4% | 27% |
|  | **Eggs** | 3.6% | 1.17 | 16% | 2.6% | 4.6% | 27% |
|  | **Cereals** | 6.5% | 1.17 | 16% | 4.7% | 8.3% | 27% |
|  | **Fruits** | 14.0% | 1.17 | 16% | 10.2% | 17.8% | 27% |
|  | **Vegetables** | 19.3% | 1.17 | 16% | 14.0% | 24.5% | 27% |
|  | **Potatoes** | 10.0% | 1.17 | 16% | 7.3% | 12.7% | 27% |
|  | **Sugarbeets** | 5.0% | 1.17 | 16% | 3.6% | 6.4% | 27% |
|  | **Oil crops** | 3.0% | 1.17 | 16% | 2.2% | 3.8% | 27% |

**Table S18:** Uncertainty factors, coefficient of variation (CV, %), lower and upper values for the waste coefficient and the variation of the limits relatively to the value of the coefficient used at retail.

|  | **Coeff** | **Unc. Factor** | **CV** | **Coeff. Lower value** | **Coeff. Upper value** | **Variation** |
| --- | --- | --- | --- | --- | --- | --- |
| **Meat** | 3.8% | 1.20 | 18% | 2.6% | 5.0% | 32% |
| **Fish** | 3.6% | 1.27 | 25% | 2.1% | 5.1% | 43% |
| **Dairy** | 0.7% | 1.27 | 25% | 0.4% | 1.0% | 43% |
| **Eggs** | 1.4% | 1.27 | 25% | 0.8% | 2.0% | 43% |
| **Rice** | 0.3% | 1.27 | 24% | 0.2% | 0.4% | 42% |
| **Household flour** | 0.5% | 1.27 | 24% | 0.3% | 0.7% | 42% |
| **Bread** | 4.8% | 1.27 | 24% | 2.8% | 6.8% | 42% |
| **Pastry** | 4.8% | 1.27 | 24% | 2.8% | 6.8% | 42% |
| **Biscuits** | 0.5% | 1.27 | 24% | 0.3% | 0.7% | 42% |
| **Pasta** | 0.5% | 1.27 | 24% | 0.3% | 0.7% | 42% |
| **Beer** | 0.5% | 1.27 | 24% | 0.3% | 0.7% | 42% |
| **Breakfast cereals** | 0.5% | 1.27 | 24% | 0.3% | 0.7% | 42% |
| **Fresh fruits** | 2.7% | 1.14 | 13% | 2.1% | 3.3% | 23% |
| **Processed fruits** | 0.4% | 1.27 | 24% | 0.2% | 0.6% | 42% |
| **Fresh vegetables** | 1.9% | 1.14 | 13% | 1.5% | 2.4% | 23% |
| **Processed vegetables** | 0.2% | 1.27 | 24% | 0.1% | 0.3% | 42% |
| **Fresh potatoes** | 2.0% | 1.13 | 12% | 1.6% | 2.4% | 21% |
| **Processed potatoes** | 0.0% | 1.13 | 12% | 0.0% | 0.0% |  |
| **Sugarbeets** | 3.3% | 1.27 | 24% | 1.9% | 4.7% | 42% |
| **Oil crops** | 0.8% | 1.27 | 24% | 0.5% | 1.1% | 42% |

**Table S19:** Uncertainty factors, coefficient of variation (CV, %), lower and upper values for the waste coefficient and the variation of the limits relatively to the value of the coefficient used at household.

| **Food Group** | **Coeff** | **Unc. Factor** | **CV (%)** | **Coeff. Lower value** | **Coeff. Upper value** | **Variation** |
| --- | --- | --- | --- | --- | --- | --- |
| **Meat** | 19% | 1.15 | 14% | 14.6% | 24.0% | 24% |
| **Fish** | 12% | 1.15 | 14% | 9.1% | 14.9% | 24% |
| **Dairy** | 8% | 1.15 | 14% | 6.3% | 10.3% | 24% |
| **Eggs** | 23% | 1.15 | 14% | 17.4% | 28.6% | 24% |
| **Rice** | 6% | 1.15 | 14% | 4.5% | 7.5% | 24% |
| **Household flour** | 6% | 1.15 | 14% | 4.5% | 7.5% | 24% |
| **Bread** | 29% | 1.15 | 14% | 21.9% | 36.1% | 24% |
| **Pastry** | 15% | 1.15 | 14% | 11.4% | 18.6% | 24% |
| **Biscuits** | 4% | 1.15 | 14% | 3.0% | 5.0% | 24% |
| **Pasta** | 6% | 1.15 | 14% | 4.5% | 7.5% | 24% |
| **Beer** | 6% | 1.15 | 14% | 4.2% | 6.8% | 24% |
| **Breakfast cereals** | 10% | 1.15 | 14% | 7.6% | 12.4% | 24% |
| **Fresh fruits** | 30% | 1.14 | 13% | 22.9% | 36.4% | 23% |
| **Processed fruits** | 11% | 1.22 | 20% | 7.2% | 14.8% | 35% |
| **Fresh vegetables** | 31% | 1.14 | 13% | 23.9% | 38.1% | 23% |
| **Processed vegetables** | 12% | 1.22 | 20% | 7.8% | 16.2% | 35% |
| **Fresh potatoes** | 30% | 1.14 | 13% | 23.4% | 37.2% | 23% |
| **Processed potatoes** | 13% | 1.15 | 14% | 9.8% | 16.2% | 24% |
| **Sugarbeets** | 12% | 1.13 | 12% | 9.5% | 14.5% | 21% |
| **Oil crops** | 16% | 1.22 | 20% | 10.4% | 21.6% | 35% |

**Table S20:** Uncertainty factors, coefficient of variation (CV, %), lower and upper values for the waste coefficient and the variation of the limits relatively to the value of the coefficient used at food service.

| **Food Group** | **Coeff** | **Unc. Factor** | **CV (%)** | **Coeff. Lower value** | **Coeff. Upper value** | **Variation** |
| --- | --- | --- | --- | --- | --- | --- |
| **Meat** | 26% | 1.55 | 46% | 5.3% | 46.5% | 80% |
| **Fish** | 22% | 1.55 | 46% | 4.5% | 40.1% | 80% |
| **Dairy** | 7% | 1.55 | 46% | 1.4% | 12.2% | 80% |
| **Eggs** | 36% | 1.55 | 46% | 7.3% | 64.7% | 80% |
| **Rice** | 18% | 1.55 | 46% | 3.7% | 32.3% | 80% |
| **Household flour** | 6% | 1.15 | 14% | 4.5% | 7.5% | 24% |
| **Bread** | 20% | 1.55 | 46% | 4.1% | 35.9% | 80% |
| **Pastry** | 20% | 1.55 | 46% | 4.1% | 35.9% | 80% |
| **Biscuits** | 4% | 1.15 | 14% | 3.0% | 5.0% | 24% |
| **Pasta** | 18% | 1.55 | 46% | 3.7% | 32.3% | 80% |
| **Beer** | 6% | 1.15 | 14% | 4.2% | 6.8% | 24% |
| **Breakfast cereals** | 10% | 1.15 | 14% | 7.6% | 12.4% | 24% |
| **Fresh fruits** | 28% | 1.55 | 46% | 5.7% | 50.7% | 80% |
| **Processed fruits** | 11% | 1.55 | 46% | 2.2% | 19.6% | 80% |
| **Fresh vegetables** | 31% | 1.55 | 46% | 6.3% | 55.9% | 80% |
| **Processed vegetables** | 9% | 1.55 | 46% | 1.9% | 16.7% | 80% |
| **Fresh potatoes** | 31% | 1.55 | 46% | 6.2% | 55.0% | 80% |
| **Processed potatoes** | 9% | 1.55 | 46% | 1.9% | 16.7% | 80% |
| **Sugarbeets** | 13% | 1.55 | 46% | 2.7% | 23.9% | 80% |
| **Oil crops** | 19% | 1.55 | 46% | 3.8% | 33.2% | 80% |

### 6.2 Contribution to variance

The contribution to variance was calculated using global sensitivity analysis. Spearman’s rank correlation coefficients (SRCC) (Hamby, 1994) was used as the measure of sensitivity that allows ranking different uncertain inputs based on their level of contributions to the variance of modelled output. Table S21 shows the coefficients contributing the most to the uncertainty of the food waste calculated for each food group and total.

**Table S21:** Contribution to variance of the coefficients for each food group

| **Food  Group** | **Coefficient stage** | **Contribution to variance** | **Food  Group** | **Coefficient** | **Contribution to variance** |
| --- | --- | --- | --- | --- | --- |
| **Meat** | Household | 61% | **Fish** | Food service | 82% |
|  | Food service | 34% |  | Household | 17% |
|  | Retail | 3% |  | Primary production (ffw) | 0.2% |
| **Dairy** | Household | 79% | **Eggs** | Household | 52% |
|  | Food service | 16% |  | Food service | 43% |
|  | Retail | 2% |  | Primary production (ffw) | 3% |
| **Cereals** | Household, Bread | 67% | **Fruit** | Primary production (ffw) | 69% |
|  | Food service, Bread | 9% |  | Household, Fresh fruits | 13% |
|  | Primary production (ffw) | 5% |  | Household, processed fruits | 7% |
| **Vegetables** | Primary Production (ffw) | 57% | **Potatoes** | Household, fresh potatoes | 65% |
|  | Household, Fresh vegetables | 27% |  | Food service, fresh potatoes | 23% |
|  | Food service, Fresh vegetables | 10% |  | Primary production (ffw) | 7% |
| **Sugarbeets** | Primarty production (ffw) | 81% | **Oilcrops** | Household | 91% |
|  | Household | 7% |  | Primary production (ffw) | 4% |
|  | Food service | 8% |  | Food service | 4% |
| **Total** | Primary production (ffw), Vegetables | 29% |  |  |  |
|  | Primary production (ffw), Fruit | 19% |  |  |  |
|  | Household, Fresh vegetables | 13% |  |  |  |

## References

Agra CEAS Consulting, 2008. The EU egg production sector, final report for Euro Group.

Beretta, C., Stoessel, F., Baier, U., Hellweg, S., 2013. Quantifying food losses and the potential for reduction in Switzerland. Waste Manag. 33, 764–773. https://doi.org/10.1016/j.wasman.2012.11.007

Clune, S., Crossin, E., Verghese, K., 2017. Systematic review of greenhouse gas emissions for different fresh food categories. J. Clean. Prod. 140, 766–783.

De Laurentiis, V., Corrado, S., Sala, S., 2018. Quantifying household food waste of fresh fruit and vegetables in the EU. Waste Manag. 77, 238–251.

EEA, 2017. Food in a green light. A systems approach to sustainable food. European Environmental Agency Report no 16/2017. https://doi.org/10.2800/884986

EUMOFA, 2014. European market observatory for fisheries and aquaculture products. 2014 Edition: The EU fish market.

EUROSTAT, 2011. Prodcom - statistics on the production of manufactured goods URL http://ec.europa.eu/eurostat/web/prodcom/ (accessed October 2017).

FAO, 1990. Handbook of fishery statistics. Indicative factors for converting product weight to live weight for a selection of major fishery commodities. Food and Agriculture Organization of the United Nations. Rome.

FAO, 2003. Technical conversion factors for agricultural commodities. Food and Agriculture Organization of the United Nations. Rome.

FAO, 2011a. FAOSTAT Commodity Balances - Livestock and Fish Primary Equivalent http://www.fao.org/faostat/en/#data/BL (accessed May 2019).

FAO, 2011b. FAOSTAT Trade statistics http://www.fao.org/faostat/en/#data/TP (accessed May 2019).

FAO, 2011c. FAOSTAT Livestock primary http://www.fao.org/faostat/en/#data/QL (accessed May 2019).

FAO, 2011d. FAOSTAT Commodity Balances - Crops Primary Equivalent [http://www.fao.org/faostat/en/#data/BC (accessed May 2019).

FEDIOL, 2013. Food, Feed and Fuels -- A Deeper Look, 2012-’13, www.fediol.eu (accessed May 2019).

Hamby, 1994. A review of techniques for parameter sensitivity analysis of environmental models. Environ. Monit Assess 32, 135–154.

Hartikainen, H., Mogensen, L., Svanes, E., Franke, U., 2018. Food waste quantification in primary production – The Nordic countries as a case study. Waste Manag. 71, 502–511. https://doi.org/10.1016/j.wasman.2017.10.026

Herbstreith, Fox, 2018. Jams, Jellies and Marmelades. http://www.herbstreith-fox.de/fileadmin/tmpl/pdf/broschueren/Konfituere_englisch.pdf (accessed March 18).

Jackson, A., Newton, R., 2016. Project to model the use of fisheries by-products in the production of marine ingredients with special reference to omega-3 fatty acids EPA and DHA. A report by IFFO and the University of Stirling. IFFO, London.

Kemna, R., van Holsteijn, F., Lee, P., Sims, E., 2017. Optimal food storage conditions in refrigeration appliances. Preparatory/review study on Commission Regulation (EC) No. 643/2009 and Commission Delegated Regulation (EU) No. 1060/2010 – complementary research on Optimal food storage conditions in refrigerator.

Leoni, C., 1997. Gli scarti dell’industria di trasformazione del pomodoro: un contributoper districarsi fra pomodoro di scarto, scarto assegnato e scarto di lavora zione. Ind. Conserv. 72, 278–290.

Marwaha, R.S., Pandey, S.K., Kumar, D., Singh, S.V., Kumar, P., 2010. Potato processing scenario in India: industrial constraints, future projections, challenges ahead and remedies—a review.". J. Food Sci. Technol. 47, 137–156.

Mena, C., Terry, L.A., Williams, A., Ellram, L., 2014. Causes of waste across multi-tier supply networks: Cases in the UK food sector. Int. J. Prod. Econ. 152, 144–158.

Muller, S., Lesage, P., Ciroth, A., Mutel, C., B.P., W., S., S., 2014. The application of the pedigree approach to the distributions foreseen in ecoinvent v3. Int. J. Life Cycle Assess. 21, 1327–1337. https://doi.org/10.1007/s11367-014-0759-5

Rickard, B., Sumner, D., 2006. EU support reductions would benefit California tomato growers and processors. Calif. Agric. 60, 207–210.

Rimestad, A.H., Løken, E.B. and Nordbotten, A., 2000. The Norwegian food composition table and the database for nutrient calculations at the Institute for Nutrition Research. Nor J Epidemiol, 10(1), pp.7-16.Roland S.R, 1988. Research note: egg shell problems: estimates of incidence and economic impact. Poultry Science, 67(12), pp.1801-1803.

Somsen, D., 2004. Production yield analysis food processing. Applications in the french-fries and the poultry-processing industries. phD Thesis, Wageningen University, Wageningen.

Sonesson, U., Davis, J., Ziegler, F., 2010. Food Production and Emissions of Greenhouse Gases: An overview of the climate impact of different product groups. Swedish Institute for food and biotechnology, Gothenburg.

Waterman, J.J., 2001. Measures, Storage Rates and Yields of Fishery Products. Torry Advisory Note 17, Department of Scientific and Industrial Research. FAO in partnership with the Support Unit for International Fisheries and Aquatic Research (SIFAR).

Willersinn, C., Mack, G., Mouron, P., Keiser, A., Siegrist, M., 2015. Quantity and quality of food losses along the Swiss potato supply chain: Stepwise investigation and the influence of quality standards on losses. Waste Manag. 46, 120–132.

WRAP, 2014. Household food and drink waste: a product focus. The Waste and Resources Action Programme, UK.

WRAP, 2011. Resource maps fruit and vegetables (RSC-008), The Waste and Resources Action Programme, UK.

WRAP, 2009. Household food and drink waste in the UK. Wastes & Resources Action Programme, UK.

Xue, L., Liu, G., Parfitt, J., Liu, X., Van Herpen, E., Stenmarck, Å., O’Connor, C., Östergren, K., Cheng, S., 2017. Missing Food, Missing Data? A Critical Review of Global Food Losses and Food Waste Data. Environ. Sci. Technol. 51, 6618–6633. https://doi.org/10.1021/acs.est.7b00401
